# Brazil nut (*Bertholletia excelsa*) and metformin abrogate cardiac complication in fructose/STZ-induced type 2 diabetic rats by attenuating oxidative stress and modulating the MAPK-mTOR/NFkB/IL-10 signaling pathways

**DOI:** 10.29219/fnr.v68.10749

**Published:** 2024-08-20

**Authors:** Zhenzuo Li, Baolan Wang, Dongfang Bai, Li Zhang

**Affiliations:** 1 Department of Endocrinology, The Fourth People’s Hospital of Jinan, Jinan, China; 2 Department of Endocrinology, Taian City Central Hospital, Taian, China

**Keywords:** *diabetes mellitus*, Bertholletia excelsa, *MAPK-mTOR*, *metabolic complication*, *cardiovascular diseases*, *heart disease*

## Abstract

**Background:**

The global prevalence of diabetic heart complication has been on the increase, and some of the drugs that are currently used to treat diabetes mellitus (DM) have not been able to mitigate this complication.

**Objective:**

This study determines the effect of Brazil nut (*Bertholletia excelsa*) and metformin on diabetic cardiomyopathy (DCM) in fructose/streptozotocin (STZ)-induced type 2 diabetic rats and also characterizes using Gas Chromatography Mass Spectrophotometry and Fourier Transform Infrared the bioactive compounds in 50% aqueous ethanol extract of Brazil nut.

**Design:**

After inducing type 2 DM, 30 male albino Wistar rats were separated into five groups that comprised of six rats per group, and they were treated as follows: groups 1 (Control) and 2 (Diabetic control) rats received rat pellets and distilled water; group 3 (Diabetic + Brazil nut) received rat pellets and Brazil nut extract (100 mg/kg, orally) dissolved in distilled water, group 4 (Diabetic + metformin) received metformin (100 mg/kg, orally) dissolved in distilled water, while group 5 (Diabetic + Brazil nut + metformin) received oral administrations of Brazil nut (100 mg/kg) and metformin (100 mg/kg) dissolved in distilled water. This study lasted for 6 weeks. The dose of Brazil nut used was selected from our pilot study on the minimum therapeutic dose of different concentrations of Brazil nut extract.

**Results:**

STZ administration induced insulin resistance, hyperglycemia, loss of weight, dyslipidemia, oxidative stress, inflammation, apoptosis, alteration of mammalian target of rapamycin, mitogen-activated protein kinase, heart function markers (creatine kinase MB, lactate dehydrogenase, and aspartate amino transaminase), and heart histology of the diabetic control, which was ameliorated after treatment with Brazil nut and metformin, but their combined treatment was better than the single treatments.

**Conclusion:**

This study shows that Brazil nut contains several bioactive compounds that support its biological properties as well as its candidature as a complementary therapy to metformin in mitigating cardiac complications arising from DM in rats.

## Popular scientific summary

- Brazil nut mitigated STZ induced pathological changes in the heart of rats but its combination with metformin were better than the single treatments especially in glycemic control
- Current study reveals the prospects of Brazil nut as a promising complementary therapy to metformin in achieving glycemic control and attenuating cardiac complication arising from diabetes mellitus in rats.

Diabetes mellitus (DM) is a serious metabolic disorder of the 21st century that has evolved into a global pandemic due to its associated morbidity and mortality (1, 2). One of the organs that is affected by prolonged DM is the heart (3). Prolonged DM triggers the development of diabetic cardiomyopathy (DCM) in the absence of atherosclerosis and hypertension by altering the structure and functions of the heart. DCM has been implicated in heart failure in diabetic patients (4–6).

Although metformin and other interventions such as acarbose, sulfonylurea, thiazolidinedione, dipeptidyl peptidase 4 (DPP4) inhibitor, etc. are currently used to treat type 2 diabetes mellitus (T2DM), many patients with T2DM progress to develop DCM, and this has been associated with diabetes-related deaths globally. In fact, earlier studies reported that 65–70% of diabetic patients died of cardiomyopathy in the last two decades, making it the major cause of mortality for both type 1 and type 2 diabetic subjects (7). The increasing global prevalence of DCM has, therefore, drawn the attention of researchers to the need for concerted efforts on ways of preventing or mitigating it.

While the pathogenesis of DCM has been reported to be multifactorial, studies have provided an insight into the progression from DM to the development of DCM to be: prolonged exposure of the heart to hyperglycemia leads to advanced glycation end products (AGE) generation, hyperinsulinemia (due to insulin resistance), and hyperlipidemia (as a result of increased mobilization of free fatty acids). Increased AGE formation and peroxidation of lipids in the myocardium lead to oxidative stress. Increased oxidative stress triggers an inflammatory state due to nuclear factor kappa B (NFkB) activation that activates other proinflammatory mediators. Oxidative stress also activates the apoptotic and renin angiotensin aldosterone signaling pathways as well as causes cardiac hypertrophy. The pathological changes lead to myocardial fibrosis, endothelial dysfunction, and, eventually, cardiomyopathy in diabetic patients (3, 5, 6).

Prolonged hyperglycemia induces reactive oxygen species (ROS) generation in the myocardium as stated earlier, altering the balance between the proapoptotic and antiapoptotic members of the Bcl-2 family, inducing apoptosis in the myocardium with resultant loss of cardiomyocytes (8). The mammalian target of rapamycin (mTOR) is a serine/threonine protein kinase that controls the growth of cells and homeostasis. Hyperglycemia-mediated activation of mTOR in the cardiovascular system has also been associated with cardiac and vascular remodeling and dysfunction that lead to DCM due to decreased insulin signaling (9). Furthermore, emerging evidence has also connected hyperglycemia-instigated aberrant activation of P38 mitogen-activated protein kinase (P38 MAPK) with cardiac and vascular remodeling and dysfunction, while the inhibition of P38 MAPK prevents the development of DCM (3). MAPK activation under diabetic condition was linked with increased oxidative stress and inflammation (10, 11). Given that antioxidants have the capacity to mitigate oxidative stress and chronic inflammation due to their electron donating ability, there has been an increased research effort in the use of natural products from plants to treat diseases that are associated with oxidative stress and inflammation.

Nuts have been identified as rich sources of antioxidants, phenolic compounds with antioxidant, and anti-inflammatory properties that could be of benefit in mitigating several diseases that are linked to oxidative stress and inflammation (12).

Brazil nut (*Bertholletia excelsa*. Humb. & Bonpl.) belongs to the tree of the Amazon Rainforest. The nut is widely found in South American countries, such as Bolivia, Brazil, Peru, South-Eastern Colombia, Guayana, Southern Venezuela, and in other countries of the world (13). Brazil nut is known to be a very good source of selenium, polyunsaturated fatty acids, and vitamins (12, 13). A few of the pharmacological properties that have been ascribed to Brazil nut include antioxidant and anti-inflammatory properties (13), and antiproliferative (14) and antihypertensive properties (13). Although the antidiabetic property of Brazil nut has also been reported, its potential benefit in preventing or treating DCM has not been reported (12).

Metformin is a biguanide and the first-choice drug for T2DM treatment due to its commendable blood glucose-lowering effect, the low risk of hypoglycemia, and decreased tendency to gain weight, which have been associated with its usage (15, 16). Although metformin was suggested to have the potential to reduce the risk of developing cardiovascular diseases in diabetic subjects, this is yet to be confirmed (16) as while some studies reported that metformin reduced the blood pressure of some animals and human subjects with DM, preventing the development of microvascular complications (15, 17–20), other studies found no effect on blood pressure reduction with metformin treatment in diabetic subjects (21, 22). In addition, although monotherapy with metformin decreases the risk of hypoglycemia, it is reportedly unable to achieve glycemic control (through HbA1c reduction) in prolonged treatment, thereby exposing diabetic patients to the risk of long-term diabetic complications (23). Recently, combination therapy with metformin or other antidiabetic drugs has been recommended for DM as it could achieve better glycemic control due to synergism and complementary action of combination therapies (23, 24).

Although the antidiabetic properties of Brazil nut and metformin have been reported (12, 15, 16), the possibility of combining them to achieve better glycemic control and prevent the development of DCM has not been reported in the literature. Due to these reasons, the present study sought to study the effect of combined administration of Brazil nut and metformin on oxidative stress, apoptosis, and the MAPK-mTOR/NFkB/interleukin 10 (IL-10) signaling pathways in the heart of fructose/streptozotocin (STZ)-induced type 2 diabetic rats, and to analyze the bioactive compounds in 50% aqueous ethanol extract of the Brazil nut using Gas Chromatography Mass Spectrophotometry (GCMS) and Fourier Transform Infrared Spectroscopy (FTIR).

## Materials and methods

### Chemicals

Commercial ELISA assay kits from MyBioSource (United States), Elab Science (China), and Cusabio (United States) were used for this study. Fructose and STZ were purchased from Sigma Chemical Company, United States. Other reagents and chemicals used that were not listed here were also of the highest grade.

### Plant experiment

Five hundred grams of Brazil nuts was purchased from the market. The nuts were washed, dried, and pulverized to flour (450 g). Thereafter, the flour was dissolved in 3 L of 50% aqueous ethanol and left for 24 h. On the following day, the nuts were filtered using a muslin cloth, and the filtrates were concentrated at 50^o^C in an oven, for 7 days to obtain the crude extract. The weight of the extract was 39.18 g with a percentage yield of 8.71%. One gram of the extract was analyzed for its mineral content using the inductively coupled plasma optical emission spectrometry. Another portion of the extract (2 g) was analyzed for its proximate composition using standard procedures. The remaining extract was dissolved in water and used for acute toxicity, minimum therapeutic dose, antidiabetic, GCMS, and FTIR studies. The selenium content of the extract was determined to be 4.31 ± 0.24 µg/g (Table 1).

**Table 1:** Nutrient composition of Brazil nut extract.

Nutrient	Composition
Protein	13.5
Fat	54.8
Carbohydrates	15.61
Energy value	609.64
Selenium	4.31
Sodium	0.09
Potassium	2.05
Calcium	3.98
Magnesium	1.15
Phosphorous	0.94
Iron	99.0
Zinc	108.18
Copper	55.52

Protein, fat, and carbohydrates- g/100 g; Energy value- kcal/100 g; Se, Sodium, Potassium, Calcium, Magnesium, Phosphorous, Iron, Zinc, and Copper- µg/g.

### Determination of lethal dose (LD_50_) of Brazil nut extract

The method of Lorke (25) was used. Eighteen male rats weighing between 140 and 150 g were divided into 6 groups of 3 rats each, following 7 days of acclimation to their feeds and water. The animals were orally administered different doses of the extract in increasing order of 10, 100, 1,000, 1,600, 2,900, and 5,000 mg/kg body weight. Following each administration, the rats were kept in stainless cages and monitored for signs of toxicity and for mortality within 24 h. Water and feeds were provided to the rats *ad libitum*. The LD_50_ of the extract was obtained as 2,154 mg/kg, and on the basis of the LD_50_ value, the doses of 100, 200, and 400 mg/kg were selected as the low, intermediate, and high doses, respectively, based on the OECD guideline.

### Determination of the minimum therapeutic dose of Brazil nut extract

Thirty male albino rats, aged 6 weeks old, were used for this study. Following 1 week of acclimatization, the rats were divided into two groups: control (five rats) and diabetic (25 rats). The control received rat pellets and distilled water for 14 days, while the disease group received rat pellets and 10% fructose drinking water (w/v) for 14 days initially and were subsequently given normal drinking water during the remaining period of the study. At the evening of the 14th day, the rats in the two groups were fasted overnight for 14 h, and the next day, their baseline blood glucose levels were determined. Thereafter, freshly prepared STZ solution (0.5 g dissolved in 25 mL of 0.1M fresh solution of sodium citrate buffer, pH 4.5) was given as an intraperitoneal injection to the rats in the disease group as a single dose at 40 mg/kg body weight (26, 27). Rats with fasting blood glucose readings that were equal to or higher than 200 mg/dL, which were determined 3 days after STZ administration, were regarded to have T2DM (26) and were advanced to the main study. Twenty diabetic rats were selected and were sub-divided into groups 2–5 with five rats per group, while the control group served as the first group, and the rats were treated as follows:

Group 1 (Control): Normal rats that were given rat pellets

Group 2 (Diabetic): Diabetic rats that were also given rat pellets

Group 3 (Study group 1): Diabetic rats that received rat pellets and oral administration of Brazil nut extract (100 mg/kg) dissolved in water

Group 4 (Study group 2): Diabetic rats that received rat pellets and oral administration of Brazil nut extract (200 mg/kg) dissolved in water.

**Fig. 1:**
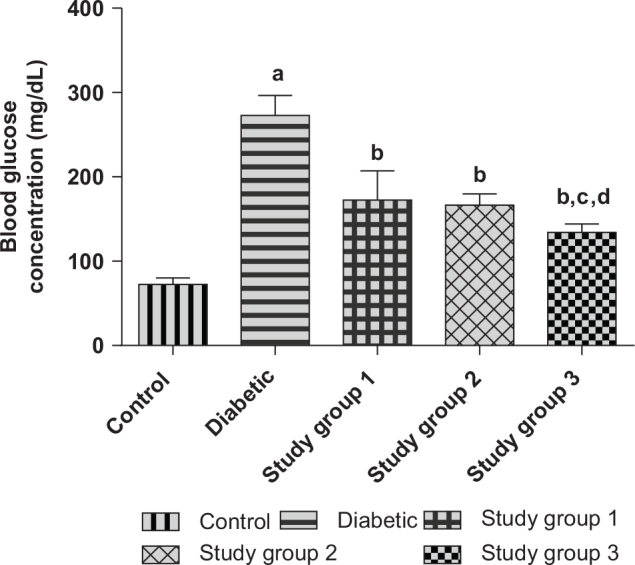
Effect of different concentrations of Brazil nut extracts on the blood glucose concentrations of rats (Pilot study). Values are reported as means ± SD. ^a^*P* < 0.05 in comparison with the control. ^b^*P* < 0.05 in comparison with the diabetic group; ^c^*P* < 0.05 in comparison with study group 1; ^d^*P* < 0.05 in comparison with study group 2. *N* = 5 rats per group. Study group 1: Diabetic + Brazil nut (100 mg/kg); Study group 2: Diabetic + Brazil nut (200 mg/kg); Study group 3: Diabetic + Brazil nut (400 mg/kg).

**Fig. 2:**
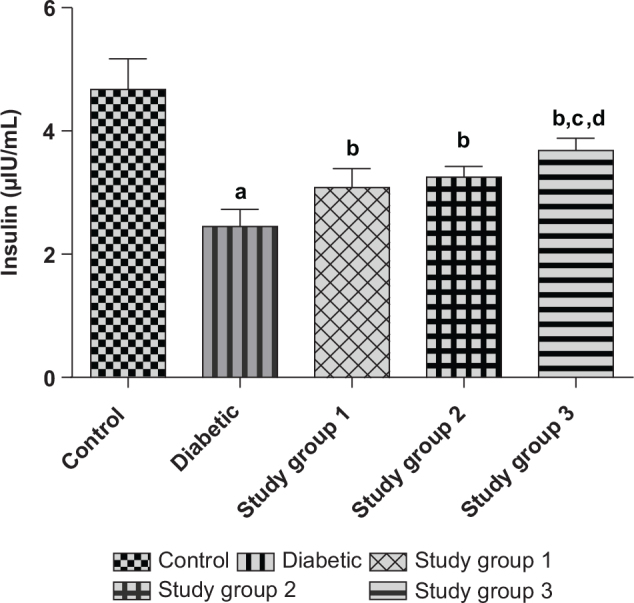
Effect of different concentrations of Brazil nut extracts on the serum insulin concentrations of rats (Pilot study). Values are reported as means ± SD. ^a^*P* < 0.05 in comparison with the control. ^b^*P* < 0.05 in comparison with the diabetic group; ^c^*P* < 0.05 in comparison with study group 1; ^d^*P* < 0.05 in comparison with study group 2. *N* = 5 rats per group. Study group 1: Diabetic + Brazil nut (100 mg/kg); Study group 2: Diabetic + Brazil nut (200 mg/kg); Study group 3: Diabetic + Brazil nut (400 mg/kg).

Group 5 (Study group 3): Diabetic rats that received rat pellets and oral administration of Brazil nut extract (400 mg/kg) dissolved in water. At the end of the period of treatment (42 days), the rats were fasted overnight, and on the next day, blood was collected from their tail vein; their blood glucose concentrations were determined using a glucometer (Acute Check). The doses that were selected for this pilot study were based on the low, intermediate, and high doses of the extract that were gotten from the LD_50_ study. The rats were thereafter sacrificed under ketamine (90 mg/kg) anesthesia, and blood was collected from their femoral veins and centrifuged (at 3,000 × g for 10 min) to obtain their sera, which were analyzed for insulin, creatine kinase myoglobin (CK-MB), and aspartate amino transaminase (AST) activities. The least dose of the extract that showed a therapeutic effect toward mitigating DM (seen as significantly decreased blood glucose and serum insulin concentrations of the study groups compared to the diabetic group) and DCM (seen as significantly decreased CK-MB and AST activities of the study groups compared to the diabetic group) was selected as the mean therapeutic dose of the extract. The nutrient composition of the rat pellets (feed) is presented in Table 2. As shown in Figs. 1, 2, and Table 3, the minimum therapeutic dose of the extract was obtained as 100 mg/kg, and it was used in the main study. Furthermore, given the Se content of the extract, this dose of the extract could provide the daily Recommended Dietary Allowance (RDA) for selenium for diabetic patients (60 kg man) as given by the National Institutes of Health (NIH), the US Department of Health and Human Services (DHHS), and the US Department of Agriculture (USDA) (12, 28, 29).

**Table 2:** Nutrient composition of rat feeds.

Nutrient	Composition
Protein	18
Fat	10
Carbohydrates	31
Crude fiber	10
Energy value	286
Selenium	0.02

Protein, fat, carbohydrates, and crude fiber- g/100 g; Energy value- Kcal/100 g; Se- µg/g.

**Table 3:** Effect of different concentrations of Brazil nut extract on some muscle function markers in the sera of fructose/STZ-induced type 2 diabetic rats (Pilot study).

Parameters	Control	Diabetic	Study group 1	Study group 2	Study group 3
CK-MB	5.02 ± 0.73	8.81 ± 0.46^a^	7.33 ± 0.66^b^	6.57 ± 0.55^b^	6.43 ± 0.74^b,c^
AST	120.32 ± 9.67	173.33 ± 5.21^a^	152.67 ± 6.72^b^	146.96 ± 7.17^b^	150.98 ± 5.62^b^

Values are reported as means ± SD.

a *P* < 0.05 in comparison with the control. Values are reported as means ± SD.

b *P* < 0.05 in comparison with the diabetic group;

c *P* < 0.05 in comparison with study group 1;

d *P* < 0.05 in comparison with study group 2. *N* = 5 rats per group. Creatine kinase MB (CK-MB) and aspartate amino transaminase (AST) – U/L. Study group 1- Diabetic + Brazil nut (100 mg/kg); Study group 2- Diabetic + Brazil nut (200 mg/kg); Study group 3- Diabetic + Brazil nut (400 mg/kg).

### Antidiabetic study (main study)

Forty male albino Wistar rats aged 6 weeks old and weighing between 120 and 140 g were recruited for this study. The rats were acclimatized for 1 week to their diets and environment before the commencement of this study. Ethical approval for this study was obtained from our Institutional Animal Committee with approval number of LL20230002. The rats were handled in line with the guidelines for the care and use of laboratory animals as was reported by the United States National Institute of Health (NIH Publications No. 80-23, revised in 1996).

### Induction of T2DM

Following the period of acclimatization, the rats were divided into two groups: control (6 rats) and disease (34 rats). The control group received rat pellets and distilled water for 14 days, while the disease group received rat pellets and 10% fructose drinking water (w/v) for 14 days initially and were subsequently given normal drinking water during the remaining period of the study.

At the evening of the 14th day, the rats in the two groups were fasted overnight for 14 h, and on the next day, their baseline blood glucose levels were determined. Thereafter, freshly prepared STZ solution (500 mg mixed with 25 mL of 0.1M fresh buffer solution of sodium citrate, pH 4.5) was given as an intraperitoneal injection to the rats in the disease group as a single dose at 40 mg/kg body weight. The rats that had fasting blood glucose readings ≥ 200 mg/dL, which was determined 3 days after the STZ administration, were regarded to have T2DM and were advanced to the main study. Twenty-four diabetic rats were selected and were subcategorized into groups 2–5, which comprised of six rats each, while the control group was taken as the first group, and the rats were subsequently treated as follows:

Group 1 (Control): Normal rats that were given rat pellets.

Group 2 (Diabetic control): Diabetic rats that were also given rat pellets.

Group 3 (Diabetic + Brazil nut): Diabetic rats that received rat pellets and oral administration of Brazil nut extract (100 mg/kg) dissolved in water.

Group 4 (Diabetic + metformin): Diabetic rats that received rat pellets and oral administration of metformin (100 mg/kg) dissolved in distilled water.

Group 5 (Diabetic + Brazil nut + metformin): Diabetic rats that received rat pellets and oral administration of Brazil nut extract (100 mg/kg) and metformin (100 mg/kg) dissolved in distilled water.

The choice of the dose of metformin was from previous studies (30). The daily food intakes and weekly body weights of the rats were measured throughout the duration of the animal study. At the end of the treatment period (6 weeks) and after an overnight fast, their body weights were measured. Blood samples were collected from their tail vein, and their blood glucose concentrations were determined using a glucometer (Acute Check). The rats were subsequently sacrificed under ketamine (90 mg/kg) anesthesia, and blood was collected from their femoral veins. The collected blood samples were divided into two portions: the first portion was put into plain tubes and centrifuged at 2,000 × g for 20 min, to get the sera, which was analyzed for C-reactive protein (CRP), CK-MB, lactate dehydrogenase (LDH), AST, total cholesterol, triacylglycerol, low-density lipoprotein cholesterol (LDL), very low-density lipoprotein cholesterol, and insulin concentration. The second portion was put into heparin tubes for HbA1C assay. The hearts of the rats were collected, blotted, weighed, and dissected into two portions: one portion was fixed in 10% formalin and used for histology studies, while the other portion was used for lipid peroxidation, antioxidant, anti-inflammatory, MTOR, p-38 MAPK, apoptotic markers (Bcl-2 and caspase-3), and total protein assays.

### Histology of the heart

The excised heart tissues were processed for histology, which consisted of embedding in molten paraffin, cooling to form blocks of paraffin, sectioning at 5 µm and hematoxylin, and eosin (H&E) staining for viewing under a light microscope. Pictures of the hearts were taken at × 400 using a light microscope.

#### Determination of serum insulin, insulin resistance, and blood levels of HbA1C

Assay for serum insulin assay was done using insulin assay kits (Monobind Inc). The values were reported as µU/mL. The homeostasis model assessment of insulin resistance (HOMA-IR) was calculated using this formula: HOMA-IR = [Serum insulin (µU/mL) × fasting blood glucose (mg/dL)/405] (31, 32). Assay for HbA1C concentrations in the whole blood of the rats was carried out using the method of Karl et al. (33).

#### Assay for inflammatory markers in the serum and heart

The CRP levels in the sera of the rats were measured using CRP assay kits obtained from Monobind Inc. (34). The results were reported as ng/mL. Assay for NF-kB and IL-10 concentrations in the hearts of the rats was done using Rat’s NFkB and IL-10 ELISA assay kits (Elabscience), following the manufacturer’s instructions. Results obtained were expressed as picogram/mg protein.

#### Determination of serum levels of heart muscle function markers

Serum activities of CK-MB were measured using CK-MB ELISA kit (CUSABIO, USA), and results were reported as U/L. LDH and AST activities in the rats’ sera were determined using Agappe assay kits (35, 36), and results were reported as U/L.

#### Determination of lipid profile in the sera

The serum concentrations of total cholesterol, TAG, and HDL were determined using Randox assay kits. VLDL was calculated using the Frieldwald formula of TAG/5, while LDL was calculated as total cholesterol – (VLDL + HDL) (37).

#### Assay of lipid peroxidation and antioxidant markers in the heart

Lipid peroxidation was measured by determining the heart concentrations of malondialdehyde (MDA) using the spectrophotometric method that was described by Buege and Aust (38). Results were reported as µmol/mg protein. Superoxide dismutase (SOD) activity in the heart of the rats was investigated following the procedure of Sun and Zigma (39), and results were reported as units/mg of protein. One unit of SOD activity was expressed as µmol of epinephrine consumed/min/mg protein. Glutathione peroxidase (GPx) activity was determined using the method of Rotruckjt et al. (40). Catalase (CAT) activity in the hearts of the rats was analyzed following the procedure of Sinha (41), and the results were reported as units/mg protein. One unit of CAT activity was defined as the amount of enzyme that catalyzes the decomposition of 1 μmol of hydrogen peroxide/min/mg protein. Glutathione-S-transferase activity was determined following the procedure of Habig et al. (42), and results were reported as units/mg protein. One unit of GST activity was expressed as nmol of substrate hydrolyzed/min/mg protein. Total proteins in the hearts of the rats were determined using the method of Tietz (34).

#### Assay for apoptotic markers in the heart

The levels of Bcl-2 and caspase 3 in the hearts of the rats were measured using ELISA assay kits (Rat Bcl-2, Cusabio, USA and Rat Caspase 3 ELISA Kits-MyBioSource, USA) by following the manufacturer’s instructions. Results obtained were reported as ng/g of protein for Bcl-2 and pmol/g of protein for caspase 3.

#### Assay for MTOR and p38-MAPK concentrations in the heart

The concentrations of MTOR and p38-MAPK in the hearts of the rats were measured with ELISA assay kits (Rat mTOR and P38 MAPK, ELISA Kits-MyBioSource) by following the procedure described by their manufacturers. Results obtained were reported as pg/mg protein for MTOR and p38-MAPK.

#### GC-MS analysis of Brazil nut extract

The GCMS investigation of the bioactive molecules in the Brazil nut extract was determined by following the procedure of Ganesh and Mohankumar (43). The extract was reconstituted in the 50% aqueous ethanol and filtered using a 0.22 µm nylon filter before injection into the GC-MS chromatogram. The bioactive compounds in the extract were identified by comparing the mass spectra of the peaks of the extract with known spectra from the database of the National Institute’s Standard and Technology.

#### FTIR analysis of Brazil nut extract

For GC-FT IR analysis, a Bio-Radiation Digilab FTS-45A spectrometer that was coupled to a Bio-Radiation Tracer equipped with a liquid Nitrogen cooled narrow-band MCT detector and coupled to a HP 5,890 series II gas chromatograph was used. The samples were eluted on a J and W DB-1 column 30 m × 0.25 mm (i.d.)/0.25 *μ*m flick width with helium as stream gas (split injection mode). Deposition tip and transmission line were held above 200°C. Absorbance spectra were recorded from 4,000 to 700 cm^-1^ at a spectral resolution of 1 cm^-1^.

### Statistical analysis

Data generated were subjected to statistical analysis using the IBM statistical package for social sciences (SPSS Statistics 29, United States). Results were reported as means ± standard deviations. One-way analysis of variance followed by Duncan Multiple Range Test was used for multiple comparisons and post hoc tests. The significance of the data sets was kept at *P* < 0.05. Graphs were generated using GraphPad Prism version 9.5.1 (GraphPad Software Inc., San Diego, CA, USA).

## Results

### Effect of different doses of Brazil nut extract on the blood glucose and serum insulin concentrations of fructose/STZ-induced type 2 diabetic rats (pilot study)

Figure 1 shows the effect of different doses of Brazil nut extract on the blood glucose concentrations of fructose/STZ-induced type 2 diabetic rats. As shown in the figure, there were significant increases (*P* < 0.05) in the blood glucose concentrations of the diabetic group compared to the control group. On the contrary, the blood glucose concentrations of the study groups were significantly decreased (*P* < 0.05) compared to the diabetic group. Furthermore, the blood glucose concentrations of the rats in study group 3 were significantly decreased (*P* < 0.05) compared to groups 1 and 2.

Figure 2 shows the effect of different doses of Brazil nut extract on the serum insulin concentrations of fructose/STZ-induced type 2 diabetic rats. As shown in the figure, there were significant decreases (*P* < 0.05) in the serum insulin concentrations of the diabetic group compared to the control group. On the contrary, the serum insulin concentrations of the study groups were significantly decreased (*P* < 0.05) compared to the diabetic group. In addition, the serum insulin concentrations of the rats in study group 3 were significantly decreased (*P* < 0.05) compared to groups 1 and 2.

Table 3 shows the effect of different doses of Brazil nut extracts on the serum CK-MB and AST activities of fructose/STZ-induced type 2 diabetic rats. As shown in the table, there were significant elevations (*P* < 0.05) in the serum CK-MB and AST activities of the diabetic group compared to the control group. On the contrary, the serum CK-MB and AST activities of the study groups were significantly decreased (*P* < 0.05) compared to the diabetic group. Furthermore, the serum CK-MB activity of study group 3 was significantly decreased (*P* < 0.05) compared to study group 1.

## Main study

### Blood glucose, serum insulin levels, HOMA-IR, and whole blood HbA1C levels

Table 4 shows the effect of Brazil nut and metformin on the blood glucose, serum insulin, and HOMA-IR values of fructose/STZ-induced type 2 diabetic rats. As shown in the table, significant elevation (*P* < 0.05) was found in the blood glucose concentrations of the diabetic group compared to the control group. The blood glucose concentrations of the rats were decreased to significant levels (*P* < 0.05) in the control group compared to the diabetic group by supplementation with Brazil nut, metformin, or combination of both. In addition, the blood glucose concentrations of the diabetic rats that received Brazil nut and metformin were decreased to significant levels (*P* < 0.05) compared to the diabetic rats that received Brazil nut alone.

**Table 4:** Effect of Brazil nut and metformin on the blood glucose, serum insulin, HOMA-IR, and glycated hemoglobin levels in fructose/STZ-induced type 2 diabetic rats.

Parameters	Control	Diabetic	Diabetic + Brazil nut	Diabetic + Metformin	Diabetic + BN + Met
Glucose	66.83 ± 11.99	292.67 ± 22.82^a^	155.17 ± 15.80^b^	131.50 ± 8.09^b,c^	108.17 ± 20.79^b,c,d^
Insulin	4.08 ± 0.50	2.72 ± 0.31^a^	3.54 ± 0.68^b^	3.42 ± 0.29^b^	3.50 ± 0.40^b^
HOMA-IR	0.68 ± 0.16	2.05 ± 0.44^a^	1.37 ± 0.39^b^	1.11 ± 0.10^b^	0.94 ± 0.22^b,c^
HbA1C	4.77 ± 0.46	7.90 ± 1.20^a^	7.33 ± 0.54	7.29 ± 0.67	6.11 ± 0.59^b,c,d^

Values are reported as means ± SD.

a *P* < 0.05 in comparison with the control.

b *P* < 0.05 in comparison with the diabetic group;

c *P* < 0.05 in comparison with the Brazil nut group;

d *P* < 0.05 in comparison with the metformin group. *N* = 6 rats per group. BN: Brazil nut; Met: Metformin; Glucose: mg/dL; Insulin: µIU/mL; HOMA-IR: Homeostatic model assessment of insulin resistance; HbA1C: Glycated hemoglobin-%.

The serum insulin concentrations of the diabetic control group were decreased to significant proportions (*P* < 0.05) compared to the control group. However, the serum insulin concentrations of the rats were significantly elevated (*P* < 0.05) compared to the diabetic group when supplementation with Brazil nut, metformin, or combination of both.

Significant elevations (*P* < 0.05) were observed for the HOMA-IR values of the diabetic group compared to the control group. In contrast, the HOMA-IR values of the diabetic rats that received Brazil nut, metformin, or combination of both were decreased to significant levels (*P* < 0.05) compared to the diabetic group. Furthermore, the HOMA-IR values of the diabetic rats that received Brazil nut and metformin were significantly decreased (*P* < 0.05) compared to diabetic rats that received Brazil nut alone.

Significant elevations (*P* < 0.05) were observed for the HbA1C levels of the diabetic group compared to the control group. Although the HbA1C levels of the diabetic rats had no significant effect (*P* > 0.05) when intervention with Brazil nut or metformin, combined administration of Brazil nut and metformin decreased to significant levels (*P* < 0.05) compared to the diabetic group. The HbA1C levels of the rats decreased to significant levels (*P* < 0.05) when intervention with a combination of Brazil nut and metformin compared to either Brazil nut or metformin.

### Organ weights and body weights of rats

Table 5 presents the effect of Brazil nut and metformin on the relative heart weights and final body weights of fructose/STZ-induced type 2 diabetic rats. Although there were no significant changes (*P* > 0.05) in the absolute heart weights of the rats across the groups (Table 5), results shown in Table 3 revealed significant elevation (*P* < 0.05) in the relative heart weight of the diabetic group compared to the control group, but this elevation was declined (*P* < 0.05) following supplementation with Brazil nut, metformin, or combination of both. In addition, the relative weights of the hearts of the diabetic rats that received Brazil nut and metformin were significantly decreased (*P* < 0.05) compared to diabetic rats that received metformin alone.

**Table 5:** Effect of Brazil nut and metformin on the relative heart weights and final body weights of fructose/STZ-induced type 2 diabetic rats.

Parameters	Control	Diabetic	Diabetic + Brazil nut	Diabetic +Metformin	Diabetic + BN + Met
Heart weight (g)	0.56 ± 0.08	0.63 ± 0.09	0.54 ± 0.14	0.56 ± 0.10	0.55 ± 0.10
RHWT (g/100 g)	0.33 ± 0.04	0.55 ± 0.01^a^	0.43 ± 0.09^b^	0.48 ± 0.04^b^	0.40 ± 0.07^b,d^
Final body weight (g)	168.03 ± 19.04	114.27 ± 16.58^a^	123.22 ± 11.36	116.72 ± 10.19	135.20 ± 11.31^b,d^

Values are reported as means ± SD.

a *P* < 0.05 in comparison with the control;

b *P* < 0.05 in comparison with the diabetic group;

d *P* < 0.05 in comparison with the metformin group. *N* = 6 rats per group. BN: Brazil nut; Met: Metformin; RHWT: Relative Heart Weight.

As shown in Table 5, the final body weight of the diabetic group was significantly decreased (*P* < 0.05) compared to the control group. Although no significant change (*P* > 0.05) was observed in the final body weights of the diabetic rats that received Brazil nut or metformin compared to the diabetic group, the final body weights of the diabetic rats that received Brazil nut and metformin increased significantly (*P* < 0.05) compared to the diabetic group. Additionally, the final body weights of the diabetic rats that received Brazil nut and metformin increased significantly (*P* < 0.05) compared to the rats that received metformin alone.

### Lipid peroxidation and antioxidant markers in the heart of fructose/STZ-induced type 2 diabetic rats

Table 6 shows the effect of Brazil nut and metformin on the lipid peroxidation and antioxidant markers in the heart of fructose/STZ-induced type 2 diabetic rats. Data presented in Table 6 showed significant elevation (*P* < 0.05) in MDA concentration in the heart of the diabetic group, which was attenuated to significant levels (*P* < 0.05) after supplementing with Brazil nut, metformin, or combination of both.

**Table 6:** Effect of Brazil nut and metformin on the lipid peroxidation and antioxidant markers in the heart of fructose/STZ-induced type 2 diabetic rats.

Parameters	Control	Diabetic	Diabetic + Brazil nut	Diabetic + Metformin	Diabetic + BN + Met
MDA	0.55 ± 0.09	1.12 ± 0.21^a^	0.79 ± 0.12^b^	0.81 ± 0.12^b^	0.75 ± 0.07^b^
SOD	7.18 ± 0.82	3.94 ± 0.62^a^	6.12 ± 0.68^b,d^	5.12 ± 0.54^b^	5.94 ± 0.24^b,d^
GPx	53.39 ± 5.58	21.81 ± 3.11^a^	28.30 ± 4.38^b^	29.30 ± 5.87^b^	38.11 ± 3.04^b,c,d^
CAT	4.25 ± 0.40	2.57 ± 0.45^a^	3.50 ± 0.68^b^	3.48 ± 0.35^b^	3.52 ± 0.74^b^
GST	440.77 ± 76.69	364.32 ± 76.42	432.26 ± 72.52	380.32 ± 43.68	386.89 ± 37.33

Data are reported as means ± SD.

a *P* < 0.05 in comparison with the control;

b *P* < 0.05 in comparison with the diabetic control group;

c *P* < 0.05 in comparison with the Brazil nut group;

d *P* < 0.05 in comparison with the metformin group; *N* = 6 rats per group. BN: Brazil nut; Met: Metformin; MDA: Malondialdehyde (µmol/mg protein); SOD: Superoxide dismutase; GPx: Glutathione peroxidase; CAT: Catalase; GST: Glutathione-S-Transferase- Units/mg protein.

Significant reductions (*P* < 0.05) were observed for the SOD, GPx, and CAT activities in the heart of the diabetic group, which were reversed following supplementation with Brazil nut, metformin, or combination of both. Furthermore, the SOD activities of the diabetic rats that received Brazil nut alone and the diabetic rats that received Brazil nut and metformin were significantly elevated (*P* < 0.05) compared to the diabetic rats that received metformin alone; the GPx activity in the heart of the diabetic rats that received Brazil nut and metformin was significantly elevated (*P* < 0.05) compared to the diabetic rats that received either Brazil nut or metformin.

No significant differences (*P* > 0.05) were observed for GST activity in the hearts of all the rats across the groups.

### Serum levels of muscle function markers

Table 7 presents the effect of Brazil nut and metformin on some muscle function markers in the sera of fructose/STZ-induced type 2 diabetic rats. As shown in the table, significant elevations (*P* < 0.05) in the serum CK, LDH, and AST activities of the diabetic group were observed compared to the control group, and these elevations were declined to significant levels (*P* < 0.05) following supplementation with Brazil nut, metformin, or combination of both. Furthermore, the AST activity of the diabetic rats that received a combination of Brazil nut and metformin was significantly reduced (*P* < 0.05) compared to the diabetic rats that received either Brazil nut or metformin.

**Table 7:** Effect of Brazil nut and metformin on some muscle function markers in the sera of fructose/STZ-induced type 2 diabetic rats.

Parameters	Control	Diabetic	Diabetic + Brazil nut	Diabetic + Metformin	Diabetic + BN + Met
CK-MB	6.17 ± 0.49	8.56 ± 0.82^a^	7.38 ± 0.80^b^	7.51 ± 0.47^b^	7.41 ± 0.85^b^
LDH	148.68 ± 6.87	190.74 ± 18.21^a^	171.41 ± 12.15^b^	167.67 ± 11.20^b^	166.20 ± 8.38^b^
AST	118.89 ± 11.26	161.80 ± 12.78^a^	143.85 ± 5.92^b^	147.87 ± 6.15^b^	130.90 ± 8.43^b,c,d^

Values are reported as means ± SD.

a *P* < 0.05 in comparison with the control. Values are reported as means ± SD.

b *P* < 0.05 in comparison with the diabetic group;

c *P* < 0.05 in comparison with the Brazil nut group;

d *P* < 0.05 in comparison with the metformin group. *N* = 6 rats per group. BN: Brazil nut; Met: Metformin; CK-MB: Creatine kinase MB; LDH: Lactate dehydrogenase; AST: Aspartate amino transaminase- U/L.

### Serum lipid profile levels of fructose/STZ-induced type 2 diabetic rats

Table 8 presents the effect of Brazil nut and metformin on the serum lipid profile of fructose/STZ-induced type 2 diabetic rats. As shown in the table, significant elevation (*P* < 0.05) was observed for the total cholesterol levels of the diabetic group compared to the control group, and supplementation with Brazil nut, metformin, or combination of both decreased (*P* < 0.05) the elevated total cholesterol levels of the diabetic rats compared to the diabetic group. No remarkable change (*P* > 0.05) was observed in the TAG and VLDL concentrations of all the rats across the groups. The LDL concentration of the diabetic group was significantly elevated (*P* < 0.05) compared to the control group. Supplementation with Brazil nut, metformin, or combination of both significantly decreased (*P* < 0.05) the LDL concentrations of the rats in contrast to the diabetic group. Furthermore, the HDL concentration of the diabetic group was significantly decreased (*P* < 0.05) compared to the control group. Supplementation with Brazil nut, metformin, or combination of both significantly increased (*P* < 0.05) the HDL concentrations of the rats compared to the diabetic group. As well, the HDL concentrations of the diabetic rats that received Brazil nut and metformin were significantly elevated (*P* < 0.05) compared to the diabetic rats that received Brazil nut alone.

**Table 8:** Effect of Brazil nut and metformin on the serum lipid profile of fructose/STZ-induced type 2 diabetic rats (mg/dL).

Parameters	Control	Diabetic	Diabetic + Brazil nut	Diabetic + Metformin	Diabetic + BN + Met
TCHOL	129.81 ± 10.80	173.17 ± 14.54^a^	143.25 ± 15.40^b^	150.20 ± 6.83^b^	153.41 ± 7.49^b^
TAG	107.17 ± 38.08	114.49 ± 24.77	101.55 ± 11.75	114.45 ± 6.61	120.76 ± 32.28
VLDL	21.43 ± 7.61	22.90 ± 4.95	20.31 ± 2.35	22.89 ± 1.32	24.15 ± 6.46
LDL	66.98 ± 18.57	119.60 ± 26.10^a^	88.03 ± 12.06^b^	83.99 ± 13.51^b^	85.89 ± 14.35^b^
HDL	41.40 ± 6.07	26.52 ± 10.29^a^	34.91 ± 3.27^b^	41.00 ± 3.82^b^	43.37 ± 4.76^b,c^

Data are reported as means ± SD.

a *P* < 0.05 in comparison with the control;

b *P* < 0.05 in comparison with the diabetic control group;

c *P* < 0.05 in comparison with the Brazil nut group. *N* = 6 rats per group. BN: Brazil nut; Met: Metformin; TCHOL: Total cholesterol; TAG: Triacylglycerol; VLDL: Very low-density lipoprotein cholesterol; LDL: low-density lipoprotein cholesterol; HDL: High-density lipoprotein cholesterol.

### Serum and cardiac concentrations of inflammatory markers and total proteins

**Fig. 3:**
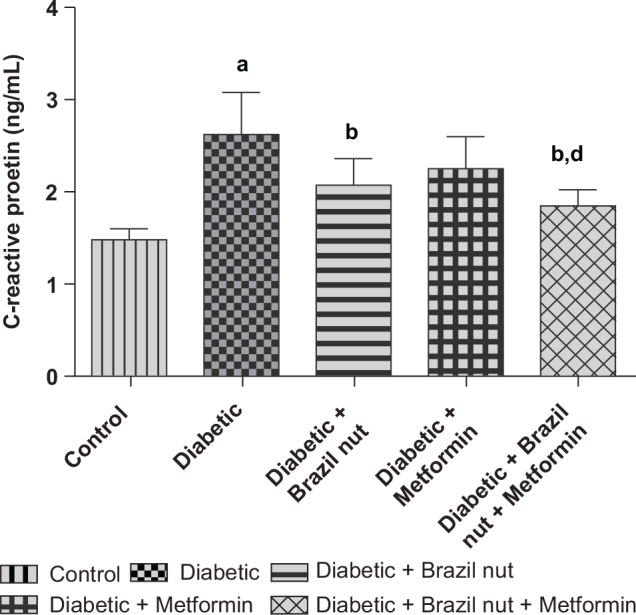
Effect of Brazil nut and metformin on C-reactive protein concentration in the sera of fructose/STZ-induced type 2 diabetic rats. Data are reported as means ± SD. ^a^*P* < 0.05 in comparison with the control; ^b^*P* < 0.05 in comparison with the diabetic control group; ^d^*P* < 0.05 in comparison with the metformin group. *N* = 6 rats per group.

Figure 3 shows the effect of Brazil nut and metformin on the serum concentrations of the inflammatory marker-CRP in fructose/STZ-induced type 2 diabetic rats. Data presented in Fig. 3 showed significant elevation (*P* < 0.05) in the serum CRP concentration of the diabetic group compared to the control group. The elevated serum CRP concentration of the diabetic group was significantly decreased (*P* < 0.05) following supplementation with Brazil nut or combination of Brazil nut and metformin, but not with metformin. As well, the CRP concentrations of the diabetic rats that received Brazil nut and metformin were significantly decreased (*P* < 0.05) compared to the diabetic rats that received metformin alone.

**Fig. 4:**
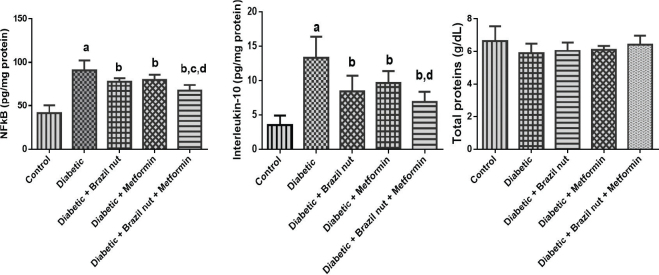
Effect of Brazil nut and metformin on inflammatory markers (NFkB -Nuclear factor kappa B and interleukin 10) and total proteins in the heart of fructose/STZ-induced type 2 diabetic rats. Data are reported as means ± SD. ^a^*P* < 0.05 in comparison with the control; ^b^*P* < 0.05 in comparison with the diabetic control group; ^c^*P* < 0.05 in comparison with the Brazil nut group; ^d^*P* < 0.05 in comparison with the metformin group. *N* = 6 rats per group. Inflammatory markers.

Figure 4 shows the effect of Brazil nut and metformin on the cardiac concentrations of some inflammatory markers (NFkB and IL-10) and total proteins in fructose/STZ-induced type 2 diabetic rats. As shown in Fig. 4, the cardiac concentration of NFkB in the diabetic group was significantly elevated (*P* < 0.05) compared to the control group, whereas the cardiac NFkB concentration of the diabetic rats that were supplemented with Brazil nut, metformin, or combination of both decreased to significant levels (*P* < 0.05) compared to the diabetic group. Furthermore, the cardiac concentration of NFkB in the diabetic rats that received Brazil nut and metformin was significantly declined (*P* < 0.05) compared to the diabetic rats that received either Brazil nut or metformin.

As well, the cardiac concentration of IL-10 in the diabetic group was significantly elevated (*P* < 0.05) compared to the control group, whereas the cardiac concentration of IL-10 in the diabetic rats that received Brazil nut, metformin, or combination of both decreased to significant levels (*P* < 0.05) compared to the diabetic group. Furthermore, the cardiac concentration of IL-10 in the diabetic rats that received Brazil nut and metformin was significantly declined (*P* < 0.05) compared to the diabetic rats that received metformin alone.

Our study found mild decreases in the total protein concentrations in the heart of the diabetic group albeit the decreases were not significant (*P* > 0.05). No significant change (*P* > 0.05) was observed in the total protein concentrations in the hearts of the diabetic rats that received Brazil nut, metformin, or combination of both compared to the diabetic group.

### Cardiac concentrations of apoptotic markers in fructose/STZ-induced type 2 diabetic rats

**Fig. 5:**
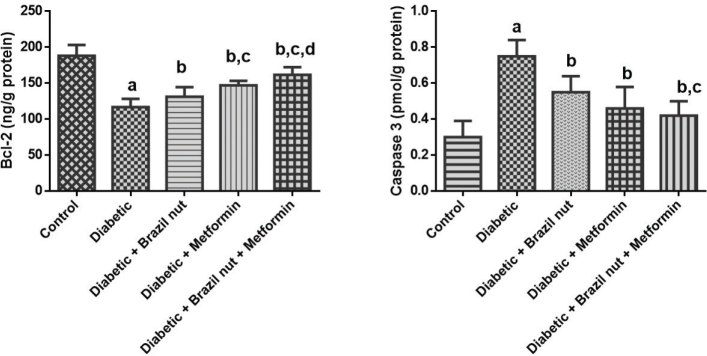
Effect of Brazil nut and metformin on the concentrations of apoptotic markers in the heart of fructose/STZ-induced type 2 diabetic rats. Data are reported as means ± SD. ^a^*P* < 0.05 in comparison with the control; ^b^*P* < 0.05 in comparison with the diabetic control group; ^c^*P* < 0.05 in comparison with the Brazil nut group; ^d^*P* < 0.05 in comparison with the metformin group. *N* = six rats per group.

Figure 5 presents the effect of Brazil nut and metformin on the cardiac concentrations of Bcl-2 and caspase 3 in fructose/STZ-induced type 2 diabetic rats. As shown in Fig. 5, the cardiac Bcl-2 concentration in the diabetic group was significantly decreased (*P* < 0.05) compared to the control group, and this decrease was reversed to significant levels (*P* < 0.05) after supplementing with Brazil nut, metformin, or combination of both. Furthermore, the Bcl-2 concentration of the diabetic rats that received metformin was significantly higher (*P* < 0.05) than that of the rats in the Brazil nut group, while the Bcl-2 concentration of the rats that received Brazil nut and metformin was significantly higher (*P* < 0.05) than the rats that received Brazil nut or metformin.

As shown in Fig. 5, the cardiac caspase 3 concentration of the diabetic group was significantly increased (*P* < 0.05) compared to the control group. The cardiac caspase 3 concentrations of the rats that supplementation with Brazil nut, metformin, or combination of both significantly decreased (*P* < 0.05) compared to the diabetic group. In addition, the caspase 3 concentration of the rats that received Brazil nut and metformin was significantly lower (*P* < 0.05) than the rats that received Brazil nut alone.

### Cardiac concentration of MTOR in fructose/STZ-induced type 2 diabetic rats

**Fig. 6:**
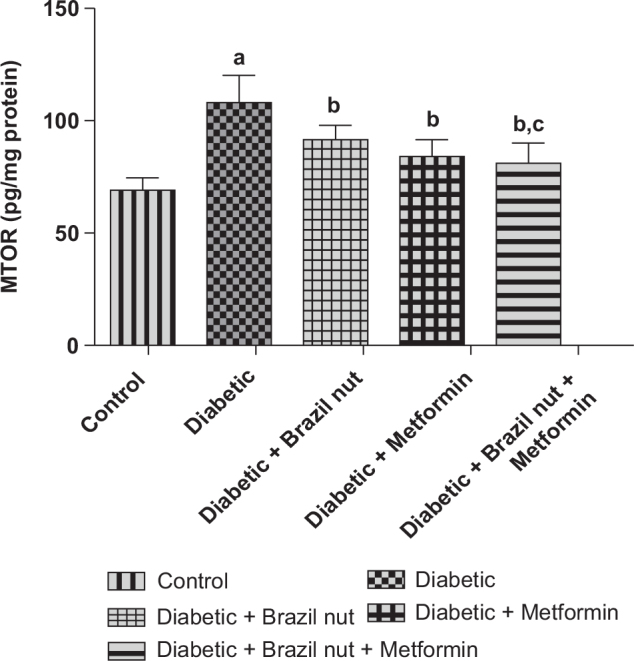
Effect of Brazil nut and metformin on the Mammalian Target of Rapamycin (MTOR) signaling pathway in the heart of fructose/STZ-induced type 2 diabetic rats. Data are reported as means ± SD. ^a^*P* < 0.05 in comparison with the control; ^b^*P* < 0.05 in comparison with the diabetic control group; ^c^*P* < 0.05 in comparison with the Brazil nut group. *N* = six rats per group.

Figure 6 shows the effect of Brazil nut and metformin on the cardiac concentration of MTOR in fructose/STZ-induced type 2 diabetic rats. As was shown in the figure, the cardiac concentration of MTOR in the diabetic group was significantly elevated (*P* < 0.05) compared to the control group, and this elevation was declined to significant levels (*P* < 0.05) following supplementation with Brazil nut, metformin, or combination of both. Furthermore, the cardiac concentration of MTOR in the diabetic rats that were given a combination of Brazil nut and metformin was significantly decreased (*P* < 0.05) in contrast to the diabetic rats that received Brazil nut alone.

### Cardiac activity of p38 MAPK in fructose/STZ-induced type 2 diabetic rats

**Fig. 7:**
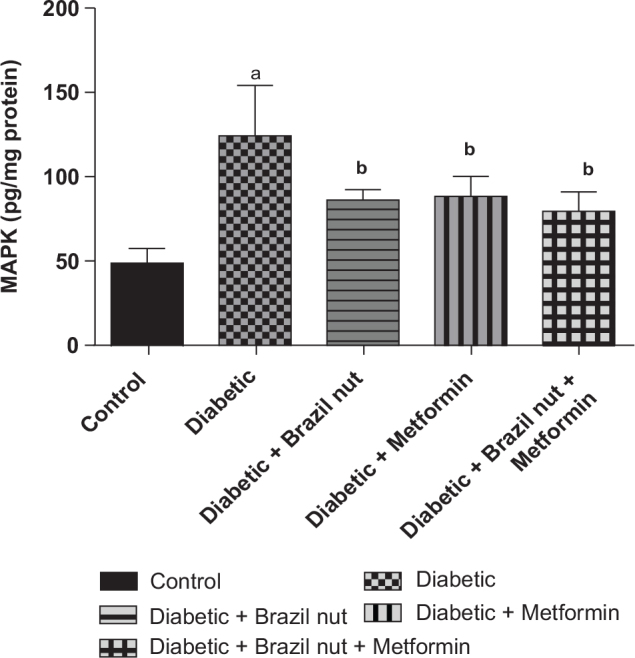
Effect of Brazil nut and metformin on MAPK signaling pathway in the heart of fructose/STZ-induced type 2 diabetic rats. Data are reported as means ± SD. ^a^*P* < 0.05 in comparison with the control; ^b^*P* < 0.05 in comparison with the diabetic control group. MAPK (P38 Mitogen Activated Protein Kinase). *N* = 6 rats per group.

Figure 7 presents the effect of Brazil nut and metformin on the cardiac activity of p38 MAPK in fructose/STZ-induced type 2 diabetic rats. As shown in Fig. 7, the cardiac p38 MAPK activity of the diabetic group was significantly elevated (*P* < 0.05) compared to the control group, and this elevation declined to significant levels (*P* < 0.05) following supplementation with Brazil nut, metformin, or combination of both.

## Histology results

**Fig. 8:**
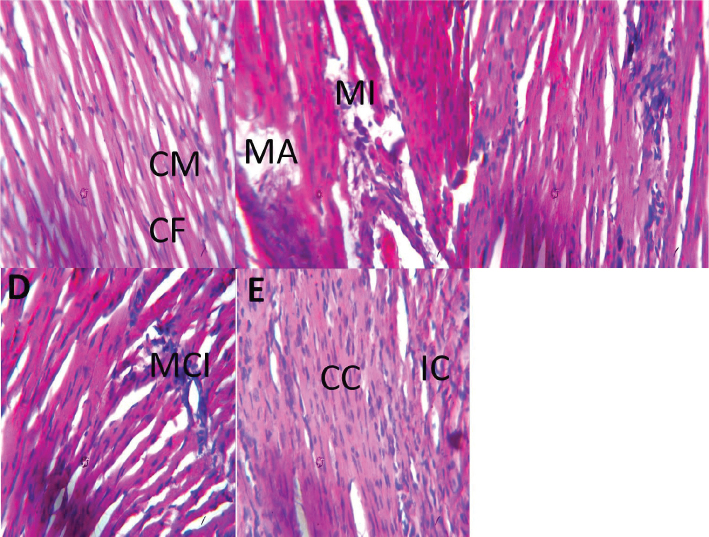
Effect of Brazil nut and metformin on the histology of the heart tissue of fructose/STZ-induced type 2 diabetic rats. (a) Histology of the muscle of the control group (x400) (H/E) shows normal cardiac tissue with well-outlined cardiac muscle (CM) and cardiac fiber (CF). (b) Histology of the cardiac muscle of the diabetic control group (x400) (H/E) shows degeneration with myocardiac inflammation (MI), myocardiac atrophy (MA), and inactive cardiac cells. (c) Histology of the cardiac muscle of the diabetic + brazil nut group (x400) (H/E) shows mild healing with moderate myocardiac inflammation (MCI). (d) Histology of the cardiac muscle of the diabetic + metformin group (x400) (H/E) shows mild healing with moderate myocardiac inflammation (MCI). (e) Histology of the cardiac muscle of the diabetic + brazil nut + metformin group (x400) (H/E) shows moderate inflammatory cells (IC) and increase in the number of active myocardiac cells (CC).

Figure 8 shows the effect of Brazil nut and metformin on the histology of the hearts of fructose/STZ-induced type 2 diabetic rats (×400) (H&E). As described in Fig. 8 (a), the heart histology of the control group (x400) (H/E) showed normal cardiac tissue with well-outlined cardiac muscle (CM) and cardiac fiber (CF). In the diabetic group (Fig. 8b), there was degeneration of their cardiac tissue with myocardiac inflammation (MI), myocardiac atrophy (MA), and inactive cardiac cells. In the diabetic + Brazil nut group (Fig. 8c), their heart histology showed mild healing of the cardiac tissue with moderate myocardiac inflammation (MI). In the diabetic + metformin group (Fig. 8d), their heart histology showed mild healing of the cardiac tissue with moderate myocardiac inflammation (MI). In the diabetic + Brazil nut + metformin group (Fig. 8e), their heart histology showed moderate inflammatory cells (MIsC) and increase in the number of active myocardiac cells (CCs).

## Plant studies

### Result of GC-MS analysis of Brazil nut extract

**Fig. 9:**
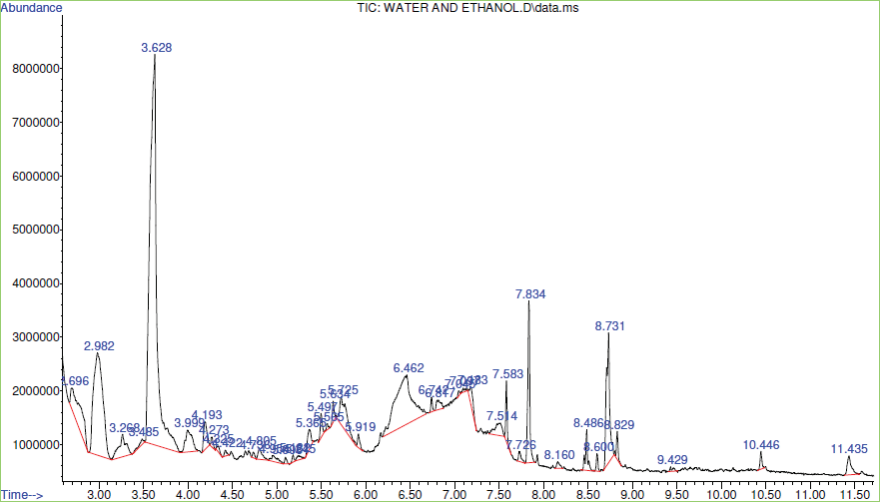
GC-MS chromatogram of Brazil nut extract.

Table 9 displays the GCMS assay results of the bioactive molecules in the 50% aqueous ethanol extract of Brazil nut and the biological activities of some identified compounds in the extract, while Fig. 9 presents the GCMS Chromatogram of the Brazil nut extract. GCMS chromatogram of the Brazil nut extract showed the presence of 40 peaks, corresponding to 40 compounds, which were identified as follows.

**Table 9:** Results of GCMS analysis of Brazil nut extract

S/N	Retention time	Name of compound	Peak area%	Class of compound	Biological activity
1	2.696	1,3-Cyclopentanedione, 2,2-dimethy 1.	3.942	Organic compound	Unknown
2	2.982	4H-Pyran-4-one, 2,3-dihydro-3,5-di hydroxy-6-methyl-	13.22	Flavonoid	Antimicrobial, antioxidant, and anti-inflammatory property (44).
3	3.268	1-Propylimidazole-2-thione	2.26	Organic compound	Unknown
4	3.485	Oxalic acid, isohexyl nonyl ester	0.36	Ester	Anti-inflammatory and antitumor properties (45–49).
5	3.628	5-Hydroxymethylfurfural	37.49	Organic compound	Antioxidant, anti-inflammatory, anti-allergic, and anti-sickling properties (50, 51)
6	3.999	1-Cyclohexene-1-carboxylic acid	2.07	Cyclic organic compound	Antimicrobial, anti-inflammatory, antioxidant properties (52, 53)
7	4.193	Octahydropyrrolo[1, 2-a]pyrazine	1.31	Phytol	Antioxidant and anti-inflammatory properties (54)
8	4.273	cis-2-Ethyl-2-hexen-1-ol	0.29	Organic compound	Unknown
9	4.325	Pentanoic acid, ethyl ester	0.21	Fatty acid ester	Flavor enhancer
10	4.422	7-Octenoic acid ethyl ester	0.19	Ester	Unknown
11	4.736	1-Ethyl-2-hydroxymethylimidazole	0.17	Imidazole	Unknown
12	4.805	3-Furanmethanol	0.63	Furan	Unknown
13	4.954	Thiazole, 5-ethenyl-4-methyl-	0.51	Thiazole	Unknown
14	5.096	Oxazole, 2,4-dimethyl-	0.18	Organic compound	Antimicrobial, anti-inflammatory, antidiabetic, anti-obesity, antioxidant properties (55)
15	5.182	2-Chloropropionic acid, hexadecyl ester	0.19	Ester	Unknown
16	5.245	Cyclopropanecarboxylic acid, 2-methyl-2-(4-methyl-3-pentenyl)-, trans-(.+-.)-	0.27	Organic compound	Unknown
17	5.365	Docosyl heptyl ether	1.01	Ester	Unknown
18	5.497	2(5H)-Furanone, 3,5,5-trimethyl-	0.73	Furanone	Unknown
19	5.565	l-(+)-Ascorbic acid 2,6-dihexadecanoate	0.17	Organic compound	Antibacterial, antioxidant, anti-inflammatory, antitumor, and wound healing properties (56, 57)
20	5.634	2,3,5,6-Tetrafluoroanisole	0.54	Organic compound	Unknown
21	5.725	6,8-Dioxa-3-thiabicyclo(3,2,1)oct e 3,3-dioxide	2.79	Organic compound	Unknown
22	5.919	Benzoic acid, 3-isothiocyanato-	0.4	Organic compound	Unknown
23	6.462	3-Morpholinopropyl isothiocyanate	11.59	Organosulfur compound	Antioxidant and anticarcinogenic (58)
24	6.742	Cyclohexane, 1,1’-oxybis-	0.33	Organic compound	Unknown
25	6.817	l-[-]-4-Hydroxy-1-methylproline	0.6	Organic compound	Unknown
26	7.045	2(5H)-Thiophenone	0.16	Organic compound	Unknown
27	7.097	Oxalic acid, monoamide, N-(3-(N-morpholinyl)propyl)-, pentyl ester	0.17	Organic compound	Unknown
28	7.183	Propan-1-one, 1-(4-ethoxyphenyl)-3 -morpholino-2-phenyl-	0.77	Organic compound	Unknown
29	7.514	3-Deoxy-d-mannoic lactone	1.26	Organic compound	Antibacterial activity (59)
30	7.583	Hexadecanoic acid, methyl ester	1.10	Ester	Antioxidant, anti-androgenic, and hypocholesterolemic properties (60)
31	7.726	Hexadecenoic acid, Z-11-	0.40	Unsaturated fatty acid	Antimicrobial properties
32	7.834	n-Hexadecanoic acid	4.58	Fatty acid	Antioxidant, anti-inflammatory, antimicrobial, anti-androgenic, hypocholesterolemic, and 5-alpha reductase inhibitory properties (61, 62)
33	8.16	Phenol, 2-chloro-4-cyclohexyl	0.28	Organic compound	Unknown
34	8.486	11-Octadecenoic acid, methyl ester,	1.22	Ester	Antidiarrhoea (63)
35	8.60	Methyl stearate	0.39	Fatty acid methyl ester	
36	8.731	Oleic acid	5.97	Monounsaturated fatty acid	Antidiabetic, antioxidant, anti-inflammatory, hypocholesterolemic, and anti-atherosclerosis properties (64–66)
37	8.829	Octadecanoic acid	0.58	Fatty acid	Hypocholesterolemic and anti-inflammatory properties (62, 67)
38	9.429	11-Hexacosyne	0.18	Alkane	
39	10.446	Hexadecanoic acid, 2-hydroxy-1-(hydroxymethyl)ethyl ester)	0.45	Ester	Antioxidant and hypercholesterolemic properties (68, 69)
40	11.435	9-Octadecenoic acid (Z)-2-hydroxy-1-(hydroxymethyl)ethyl ester	1.06	Ester	Antimicrobial, anti-inflammatory, anticancer, and diuretic properties (70)

GC-MS: Gas chromatography mass spectrophotometry.

Compound 1 was identified as 2,2-dimethy 1,3-cyclopentanedione with a retention time (RT) of 2.696 and a percentage peak area (PA) of 3.942. Compound 2 was identified as 4H-Pyran-4-one, 2,3-dihydro-3,5-di hydroxy-6-methyl- with an RT of 2.982 and a PA of 13.22. Compound 3 was identified as 1-propylimidazole-2-thione with an RT of 3.268 and a PA of 2.26. Compound 4 was identified as oxalic acid, isohexyl nonyl ester with an RT of 3.485 and a PA of 0.36. Compound 5 was identified as 5-hydroxymethylfurfural with an RT of 3.628 and a PA of 37.49. Compound 6 was identified as 1-cyclohexene-1-carboxylic acid with an RT of 3.999 and a PA of 2.07. Compound 7 was identified as octahydropyrrolo[1,2-a]pyrazine with an RT of 4.193 and a PA of 1.31. Compound 8 was identified as cis-2-ethyl-2-hexen-1-ol with an RT of 4.273 and a PA of 0.29. Compound 9 was identified as pentanoic acid, ethyl ester with an RT of 4.325 and a PA of 0.21. Compound 10 was identified as 7-octenoic acid ethyl ester with an RT of 4.422 and a PA of 0.19. Compound 11 was identified as 1-ethyl-2-hydroxymethylimidazole with an RT of 4.736 and a PA of 0.17. Compound 12 was identified as 3-furanmethanol with an RT of 4.805 and a PA of 0.63. Compound 13 was identified as thiazole, 5-ethenyl-4-methyl- with an RT of 4.954 and a PA of 0.51. Compound 14 was identified as oxazole, 2,4-dimethyl- with an RT of 5.096 and a PA of 0.18. Compound 15 was identified as 2-chloropropionic acid, hexadecyl ester with an RT of 5.182 and a PA of 0.19. Compound 16 was identified as cyclopropanecarboxylic acid, 2-met hyl-2-(4-methyl-3-pentenyl)-, trans-(+)- with an RT of 5.245 and a PA of 0.27. Compound 17 was identified as docosyl heptyl ether with an RT of 5.365 and a PA of 1.01. Compound 18 was identified as 2(5H)-furanone, 3,5,5-trimethyl- with an RT of 5.497 and a PA of 0.73. Compound 19 was identified as l-(+)-ascorbic acid 2,6-dihexadecanoate with an RT of 5.565 and a PA of 0.17. Compound 20 was identified as 2,3,5,6-tetrafluoroanisole with an RT of 5.634 and a PA of 0.54. Compound 21 was identified as 6,8-dioxa-3-thiabicyclo(3,2,1)octa ne 3,3-dioxide with an RT of 5.725 and a PA of 2.79. Compound 22 was identified as benzoic acid, 3-isothiocyanato- with an RT of 5.919 and a PA of 0.4. Compound 23 was identified as 3-morpholinopropyl isothiocyanate with an RT of 6.462 and a PA of 11.59. Compound 24 was identified as cyclohexane, 1,1’-oxybis- with an RT of 6.742 and a PA of 0.33. Compound 25 was identified as l-[-]-4-hydroxy-1-methylproline with an RT of 6.817 and a PA of 0.60. Compound 26 was identified as 2(5H)-thiophenone with an RT of 7.045 and a PA of 0.16. Compound 27 was identified as oxalic acid, monoamide, N-(3-(N-morpholinyl)propyl)-, pentyl ester with an RT of 7.097 and a PA of 0.17. Compound 28 was identified as propan-1-one, 1-(4-ethoxyphenyl)-3-morpholino-2-phenyl- with an RT of 7.183 and a PA of 0.77. Compound 29 was identified as 3-deoxy-d-mannoic lactone with an RT of 7.514 and a PA of 1.26. Compound 30 was identified as hexadecanoic acid, methyl ester with an RT of 7.583 and a PA of 1.10. Compound 31 was identified as Z-11-hexadecenoic acid with an RT of 7.726 and a PA of 0.40. Compound 32 was identified as n-hexadecanoic acid with an RT of 7.834 and a PA of 4.58. Compound 33 was identified as phenol, 2-chloro-4-cyclohexyl with an RT of 8.16 and a PA of 0.28. Compound 34 was identified as 11-octadecenoic acid, methyl ester with an RT of 8.486 and a PA of 1.22. Compound 35 was identified as methyl stearate with an RT of 8.60 and a PA of 0.39. Compound 36 was identified as oleic acid with an RT of 8.731 and a PA of 5.97. Compound 37 was identified as octadecanoic acid with an RT of 8.829 and a PA of 0.58. Compound 38 was identified as 11-hexacosyne with an RT of 9.429 and a PA of 0.18. Compound 39 was identified as 2-hydroxy-1-(hydroxymethyl)ethyl ester)hexadecanoic acid with an RT of 10.446 and a PA of 0.45, while compound 40 was identified as 9-octadecenoic acid (Z)-2-hydroxy-1-(hydroxymethyl)ethyl ester with an RT of 11.435 and a PA of 1.06.

### FTIR results of Brazil nut extract

**Fig. 10:**
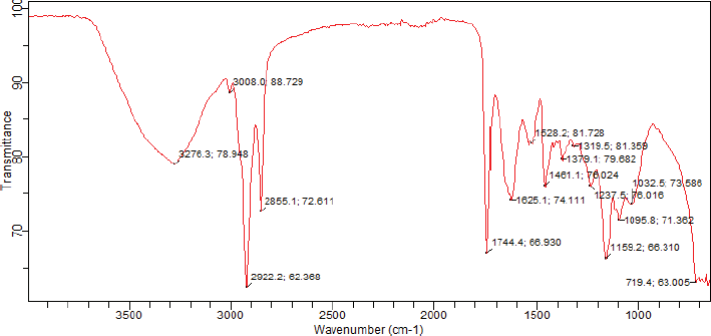
Fourier-transform infrared spectra of Brazil nut extract.

Table 10 shows the functional groups that were identified with FTIR in the Brazil nut extract, while Fig. 10 shows the FTIR spectra of the extract. FTIR analysis of the Brazil nut extract showed many peaks, representing the functional groups that are present in the extract. The band at 3,276 cm^-1^ broad shows O-H stretching of alcoholic and phenolic groups, while the band at 2,922 cm^-1^ is due to SP^3^ -C-H stretching. The absorption band at 1,744 cm^-1^ is due largely to carbonyl C = O stretching of fatty acids, ketones, and esters. The presence of strong peaks at 3,276 cm^-1^ in the FTIR assay correlates with the GCMS assay that identified the presence of alcohols, fatty acids, esters, and phenolic compounds as the major components of the extract.

**Table 10:** Functional groups identified with Fourier transform infrared spectroscopy

Extract	Wavenumber (cm^-1^)	Functional group	Compounds identified
Ethanol/water	3276.3	O-H stretching	Phenolic groups or Alcohol
	2922.2	C-H stretching	Lipids, proteins
	1744.4	C = O stretching	Aldehydes, Fatty acids, Ketones, Esters
	1625.1	C = N stretching	Amide
	1095.8	PO_2_^-^ stretching	Phosphate ion

## Discussion

This study showed that fructose/STZ administration to the diabetic group induced insulin resistance and type 2 diabetes, which were evident from their decreased serum insulin concentrations, elevated blood glucose levels, HOMA-IR, and HbA1C values compared to the control group. Previous studies showed that IR and decreased insulin secretion are central to the development of DM (71). The decreased insulin concentration and elevated levels of glucose and HOMA-IR in the diabetic rats were reversed following supplementation with Brazil nut, metformin, or combination of both, a finding that suggests the antidiabetic properties of Brazil nut or its combination with metformin. Our result corroborates earlier reports of Nascimento et al. (12) on the antidiabetic properties of Brazil nut. Furthermore, metformin demonstrated better glucose lowering properties than Brazil nut, and combined administration of Brazil nut and metformin demonstrated better glucose reduction than Brazil nut or metformin monotherapy and better attenuation of IR than Brazil nut monotherapy, and this is noteworthy.

HbA1C is an early glycation end product that is used to assess glycemic control in diabetic patients (72). Although intervention with Brazil nut or metformin did not attenuate the elevated HbA1C concentrations of the diabetic rats, combined administration of Brazil nut and metformin diminished the elevated HbA1C concentrations of the diabetic rats, suggesting better control of glycaemia with a combination of Brazil nut and metformin.

This study further showed cardiac hypertrophy (increased relative heart weight) and decreased mean body weight for the diabetic group. As a matter of fact, organ and body weights are important markers of plants or drug toxicity (11). Previous studies reported weight loss in diabetic condition (11, 73). Weight loss as obtained in the current study for the diabetic group could be attributed to diminished insulin secretion, leading to elevated muscle protein catabolism to make up for the carbohydrates that are not available to be used as an energy source (74). This study showed diminished cardiac hypertrophy following supplementation with Brazil nut, metformin, or combination of both. Furthermore, intervention with Brazil nut and metformin polytherapy impeded weight loss in the diabetic rats unlike Brazil nut or metformin monotherapy, and the rats that received a combination of Brazil nut and metformin were found to weigh better than the rats that received metformin alone. Current study therefore indicates the potential usefulness of Brazil nut as a complementary therapy to metformin, in mitigating STZ-induced cardiac toxicity and weight loss.

Increased ROS production in the heart of diabetic subjects contributes to the incidence and progression of DCM (75). This study found diabetes-instigated increased cardiac oxidative stress as evidenced from the increased heart level of the lipid peroxidation indicator-MDA but decreased SOD activity, GPx activity, and CAT activity in the diabetic heart. Previous studies showed that interventional measures that either blunt ROS generation or augmented myocardial antioxidant defense systems are of efficacy in mitigating diabetes-instigated cardiomyopathy (75, 76). Therefore, the decreased MDA concentrations but increased SOD, GPx, and CAT activities in the hearts of the diabetic rats that were supplemented with Brazil nut, metformin, or combination of both suggest the promising role of Brazil nut as a single or complementary therapy to metformin in mitigating diabetes-instigated cardiac oxidative stress. Our study further shows that Brazil nut demonstrated better SOD enhancing property than metformin, and its combination with metformin demonstrated better SOD and GPx enhancing properties than metformin. In addition, combined administration of Brazil nut and metformin demonstrated better GPx enhancing properties than Brazil nut or metformin. Current study therefore reveals that Brazil nut potentiated the antioxidant property of metformin, and its combination with metformin demonstrated better antioxidant properties than Brazil nut or metformin monotherapies.

The non-significant changes in the cardiac GST activities of all the rats across the groups might suggest the non-participation of GST in abrogation of diabetes-instigated cardiac oxidative stress in the current study.

The effect of Brazil nut and metformin on diabetes-instigated systemic inflammation was determined by measuring the serum CRP concentrations of the rats. CRP is an acute phase protein that is produced by the liver in response to inflammation, and while it is regarded as an early marker of a disease condition, increased CRP levels have also been found in chronic inflammation (77). Our current study found elevated serum CRP levels in the diabetic group, a finding that corroborates previous reports of Gupta et al. (78) and Al-Rasheed et al. (79) on elevated CRP levels in diabetic animals. Interestingly, the elevated serum CRP levels in the diabetic group were attenuated following interventions with Brazil nut or its combination with metformin (unlike metformin alone), suggesting the anti-inflammatory properties of these interventions.

Next, we determined the effect of Brazil nut and metformin on diabetes-instigated cardiac inflammation by measuring the cardiac concentrations of NFkB and IL-10 in the rats.

Chronic hyperglycemia and its accompanying ROS generation can lead to the release of pro-inflammatory cells, leading to NFkB activation (5). NFkB activation is central to the release of pro-inflammatory (80) and anti-inflammatory cytokines, including IL-10, an anti-inflammatory cytokine that is released by the interleukin family in response to inflammation (81). Therefore, the increased cardiac concentrations of NFkB and IL-10 in the diabetic control group infer diabetes-instigated cardiac inflammation. Cardiac inflammation has also been reported to promote degenerative changes in the cardiac tissue, including cardiomyocyte damage that culminates in DCM (79). The decreased cardiac concentration of NFkB and IL-10 in the diabetic rats following supplementation with Brazil nut, metformin, or combination of both suggests the protective action of Brazil nut, metformin, or combination of both against diabetes-induced cardiac inflammation. In addition, this study shows that Brazil nut and metformin combinatorial therapy demonstrated better protective action against diabetes-instigated cardiac inflammation than Brazil nut or metformin monotherapy. Our study found no significant change in the cardiac total protein concentrations of all the rats across the groups, and while this cannot be explained in this study, it is noteworthy.

CK-MB, LDH, and AST are sensitive cardiac function markers (5, 79, 82). In addition, CK-MB is a specific indicator of the impairment of the myocardium (83). These molecules are released from cardiac muscle cells into the circulation during myocardial injury or pathology, leading to their elevated serum levels (5, 79, 82, 83). Previous studies reported that during hyperglycemia, increased generation of ROS leads to alteration of the cell membrane of cardiomyocytes through interaction with their membrane components, leading to the release of CK-MB, AST, and LDH from damaged cardiac cells into the circulation (8). As such, serum levels of CK-MB, LDH, and AST have been reported to be elevated during DCM (5, 79, 82).

Our study found remarkable elevation in the serum activities of CK-MB, LDH, and AST in the diabetic group, which corroborates previous reports (8, 82, 83). This elevation might have arisen from increased membrane permeability of the cardiomyocytes and subsequent release of these enzymes from the cardiac cells into the circulation, as a result of oxidative damage to the myocardium that was brought on by fructose/STZ-induced hyperglycemia. The elevated serum activities of CK-MB, LDH, and AST were abrogated upon supplementation with Brazil nut, metformin, or combination of both. Our study further shows that a combination of Brazil nut and metformin mitigated elevated serum AST activities of the diabetic rats better than Brazil nut or metformin monotherapies. This study reveals the cardioprotective actions of these interventions in the diabetic heart, which were found to be better with the combined therapies than the monotherapies. The cardiac protective actions of these interventions as obtained in this study may not be unconnected to their antioxidant property, which could have prevented the cardiomyocytes from oxidative damage instigated by fructose/STZ.

Dyslipidemia refers to an elevation in blood concentrations of total cholesterol or LDL and decreased concentrations of HDL (Fodor, 2011). Dyslipidemia is another cause of DCM, and it is characterized by elevated circulating concentrations of TC or LDL and low levels of HDL (3). Dyslipidemia promotes the deposition of TAG and cholesterol in the myocardium, leading to diastolic dysfunction, inflammation, cardiac IR, and fibrosis (3, 79). Our results show the development of dyslipidemia in the diabetic group as seen from the elevated TC and LDL as well as the decreased HDL concentrations in the sera of this group.

Brazil nut, metformin, or combination of both mitigated diabetes-induced dyslipidemia, leading to lower TC and LDL as well as higher LDL concentrations in the diabetic rats that were treated with these interventions compared to the diabetic group.

We further showed that fructose/STZ-induced type 2 DM instigated apoptosis induction in the hearts of the diabetic group. This was evident from the decreased concentration of the antiapoptotic marker-Bcl-2 and the increased concentration of the proapoptotic marker-caspase 3 in the heart of the diabetic group relative to the control. Brazil nut, metformin, and combination of both demonstrated the capacity to blunt cardiac apoptosis induced by fructose/STZ as seen from the increased concentration of Bcl-2 but decreased concentration of caspase 3 in the hearts of the rats that received these interventions. Similar reports on the attenuation of apoptosis by Brazil nut were given by Cury et al. (84). Again, combined administration of Brazil nut and metformin was better than the single treatments in abrogating fructose/STZ-induced cardiac apoptosis, as evidenced from the increased Bcl-2 concentrations of the rats in the Brazil nut + metformin group compared to the rats in the Brazil nut or metformin group.

Compelling evidence has shown that p38-MAPK was aberrantly upregulated in experimental and clinical studies on DM, and its inhibition prevented the development of DCM (3, 85). p38-MAPK has also been suggested as a promising therapeutic target for blunting DCM (3). Furthermore, mTOR is reported to be central to the induction of cardiac hypertrophy after stress, and its inhibition has been reported to improve cardiac function during DCM (75, 86). The increased cardiac concentrations of p38 MAPK and mTOR in the diabetic group further affirm diabetes-induced myocardial changes in the diabetic group. Previous studies found that metformin inhibited MAPK signaling, inducing insulin secretion in T2DM (87), a finding that corroborates our study. Our study further demonstrates the ability of Brazil nut and metformin polytherapy to improve diabetes-induced alteration of the cardiac tissue, as seen from the reduced cardiac concentrations of p38 MAPK and MTOR in the heart of the diabetic rats that were given these interventions.

The outcome of the heart histology of the experimental rats supports the biochemical results that we obtained, further affirming the protective action of Brazil nut or its combination with metformin against diabetes-instigated cardiac complication. Combined administration of Brazil nut and metformin improved the cardiac histology of the diabetic rats better than either Brazil nut or metformin, indicating better protection against fructose/STZ-induced cardiac complication with Brazil nut and metformin than either Brazil nut or metformin. This study therefore highlights the promising potential of Brazil nut as a complementary therapy to metformin in mitigating cardiac complication arising from fructose/STZ diabetes in rats. Previous studies reported that exposure of rats to a low dose (75 mg) of Brazil nut resulted to their protective action against renal inflammation and apoptosis in a rat model of ischemia and reperfusion injury, whereas exposure of rats to 150 mg of Brazil nut exacerbated the renal expression of cyclooxygenase-2 in rats with ischemia and reperfusion injury (84). The cardiac protective action of Brazil nut as demonstrated in this study suggests that the consumption of this nut at the studied dose is necessary to maximize its protective action against diabetic cardiac complication in rats. This dose might also help to meet the daily nutritional requirement of selenium for diabetic patients since Se from Brazil nut is reported to be bioavailable with its bioavailability, comparable to that of other inorganic forms of Se (88).

Functional group identification in FTIR analysis is based on FT-IR peaks arising from stretching and bending liberations (89, 90). The GC–MS and FTIR analyses of the Brazil nut extract clearly showed the presence of alcohols, fatty acids, esters, and phenolic compounds as the principal components of the extract. The presence of these compounds in the extract with antioxidant, anti-inflammatory, and antidiabetic properties might explain the antioxidant, anti-inflammatory, and antidiabetic properties of the extract as demonstrated in this study.

In conclusion, this study shows that Brazil nut potentiated the antidiabetic property of metformin, and its combination with metformin exerted better glycemic control and protective action against diabetes-instigated cardiomyopathy than either Brazil nut or metformin. This makes Brazil nut a promising complementary therapy to metformin that could be useful in achieving glycemic control and preventing the development of DCM. Finally, this study shows that Brazil nut contains several bioactive compounds that support its biological activities as obtained in this study.

## Author contributions

Conceptualization – Z.L., B.W., D.B., and L.Z. Methodology, Z.L., B.W., D.B., and L.Z. Software – L.Z Formal analysis: L.Z. Investigation- L.Z. Data curation: L.Z. Writing – original draft preparation, L.Z. Writing – review and editing, Z.L., B.W., D.B., and L.Z. All authors gave final approval of the submitted version.

## References

[1] Tabish SA. Is diabetes becoming the biggest epidemic of the twenty-first century? Int J Health Sci 2007; 1(2): V–VIII. PMC3068646 21475425

[2] Gupta SK, Dongare S, Mathur R, Mohanty IR, Srivastava S, Mathur S, et al. Genistein ameliorates cardiac inflammation and oxidative stress in streptozotocin-induced diabetic cardiomyopathy in rats. Mol Cell Biochem 2015; 408(1–2): 63–72. doi: 10.1007/s11010-015-2483-2 26092427

[3] Fan Z, Dong J, Mub Y, Liu X. Nesfatin-1 protects against diabetic cardiomyopathy in the streptozotocin-induced diabetic mouse model via the p38-MAPK pathway. Nesfatin-1 protects against diabetic cardiomyopathy in the streptozotocin-induced diabetic mouse model via the p38-MAPK pathway. Bioengineered 2022; 13(6): 14670–81. doi: 10.1080/21655979.2022.2066748 35818327 PMC9342195

[4] Wilson AJ, Gill EK, Abudalo RA, Edgar KS, Watson CJ, Grieve DJ. Reactive oxygen species signalling in the diabetic heart: emerging prospect for therapeutic targeting. Heart 2017; 104: 293–9. doi: 10.1136/heartjnl-2017-311448 28954833

[5] Wen C, Liu C, Li Y, Xia T, Zhang X, Xue S, et al. Ameliorative potentials of the ethanolic extract from *Lycium chinense* leaf extract against diabetic cardiomyopathy. Insight into oxido-inflammatory and apoptosis modulation. Biomed Pharmacother 2022; 154: 113583. doi: 10.1016/j.biopha.2022.113583 35994819

[6] Kameshwaran S, Ravisankar M, Srinivasan P, Suresh V. Ameliorative impact of *Tecoma stans* extract on streptozotocin-induced diabetic cardiomyopathy in Wistar rats. J Pharm Pharmacol 2023; 1(1): 55–62. doi: 10.9734/bpi/acpr/v1/5974E

[7] Cai L, Kang, YJ. Oxidative stress and diabetic cardiomyopathy. Cardiovascular Toxicol 2001; 1: 181–93. doi: 10.1385/CT:1:3:181 12213971

[8] Naghdi A, Goodarzi MT, Karimi J, Hashemnia M, Khodadadi I. Effects of curcumin and metformin on oxidative stress and apoptosis in heart tissue of type 1 diabetic rats. J Cardiovasc Thorac Res 2022; 14(2): 128–37. doi: 10.34172/jcvtr.2022.23 35935389 PMC9339728

[9] Chong ZZ, Maiese K. Mammalian target of rapamycin signaling in diabetic cardiovascular disease. Cardiovas Diabetol 2012; 11: 45. doi: 10.1186/1475-2840-11-45 PMC3398846 22545721

[10] Abdel-Aziz AM, Abozaid SMM, Yousef RKM, Mohammed MM, Khalaf, KM. Fenofibrate ameliorates testicular damage in rats with streptozotocin-induced type 1 diabetes: role of HO-1 and p38 MAPK. Pharmacol Rep 2020; 72(6): 1645–56. doi: 10.1007/s43440-020-00096-0 32515004

[11] Alabi TD, Chegou NN, Brooks NL, Oguntibeju OO. Effects of *Anchomanes difformis* on inflammation, apoptosis, and organ toxicity in STZ-induced diabetic cardiomyopathy. Biomedicines 2020; 8: 29. doi: 10.3390/biomedicines8020029 32046294 PMC7168158

[12] Nascimento LPS, Pires VC, Ribeiro DA, Gollücke APB, Yamamura H, Junior OA. Benefits of the consumption of Brazil nut (*Bertholletia excelsa*) extract in male reproductive parameters of streptozotocin-induced diabetic rats. J. Diabetes Metab Dis 2020; 19(1): 187–196. doi: 10.1007/s40200-020-00490-8 PMC7270419 32550168

[13] Frausto-González O, Bautista CJ, Narváez-González F, Hernandez-Leon A, Estrada Camarena E, Rivero-Cruz F, et al. *Bertholletia excelsa* seeds reduce anxiety-like behavior, lipids, and overweight in mice. Molecules 2021; 6: 3212. doi: 10.3390/molecules26113212 PMC8198383 34072024

[14] Atlantis E, Goldney RD, Wittert GA. Obesity and depression or anxiety. BMJ 2009; 339: 871–6. doi: 10.1136/bmj.b3868 19808767

[15] Kinaan M, Ding H, Triggle CR. Metformin: an old drug for the treatment of diabetes but a new drug for the protection of the endothelium. Med Princ Pract 2015; 24: 401–15. doi: 10.1159/000381643 26021280 PMC5588255

[16] Rhee SY, Kim HJ, Ko SH, Hur KY, Kim NH, Moon MK, et al. Monotherapy in patients with type 2 diabetes mellitus. Diabetes Metab J 2017; 41: 349–56. doi: 10.4093/dmj.2017.41.5.349 29086532 PMC5663673

[17] UK Prospective Diabetes Study Group. Tight blood pressure control and risk of macrovascular and microvascular complications in type 2 diabetes (UKPDS 38). Br Med J 1998; 317(7160): 703–13. doi: 10.1136/bmj.317.7160.703 9732337 PMC28659

[18] Muntzel MS, Hamidou I, Barrett S. Metformin attenuates salt-induced hypertension in spontaneously hypertensive rats. Hypertension 1999; 33: 1135–40. doi: 10.1161/01.HYP.33.5.1135 10334800

[19] Abbasi F, Chu JW, McLaughlin T, Lamendola C, Leary ET, Reaven GM. Effect of metformin treatment on multiple cardiovascular disease risk factors in patients with type 2 diabetes mellitus. Metabolism 2004; 53(2): 159–64. doi: 10.1016/j.metabol.2003.07.020 14767866

[20] Arunachalam G, Samuel SM, Marei I, Ding H, Triggle CR. Metformin modulates hyperglycaemia-induced endothelial senescence and apoptosis through SIRT1. Br J Pharmacol 2014; 171: 523–35. doi: 10.1111/bph.12496 24372553 PMC3904269

[21] Calle-Pascual AL, Garcia-Honduvilla J, Martin-Alvarez PJ, Vara E, Calle JR, Munguira ME, et al. Comparison between acarbose, metformin, and insulin treatment in type 2 diabetic patients with secondary failure to sulfonylurea treatment. Diab Metab 1995; 21: 256–60. 8529760

[22] Gudbjornsdottir S, Friberg P, Elam M, Attvall S, Lonnroth P, Wallin G. The effect of metformin and insulin on sympathetic nerve activity, norepinephrine spillover and blood pressure in obese, insulin resistant, normoglycemic, hypertensive men. Blood Pressure 1994; 3: 394–403. doi: 10.3109/08037059409102293 7704288

[23] Singh AK, Singh R, Chakraborty PP. Diabetes monotherapies versus metformin-based combination therapy for the treatment of type 2 diabetes. Int J Gen Med 2021; 14: 3833–48. doi: 10.2147/IJGM.S295459 34335049 PMC8318007

[24] Alotaibi MR, Fatani AJ, Almnaizel AT, Ahmed MM, Abuohashish HM, Al-Rejaie SS. *In vivo* assessment of combined effects of Glibenclamide and Losartan in diabetic rats. Med Princ Pract 2019; 28: 178–85. doi: 10.1159/000496104 30537701 PMC6545916

[25] Lorke D. A new approach to practical acute toxicity testing. Arch Toxicol 1983; 54: 275–87. doi: 10.1007/BF01234480 6667118

[26] Abdel-Ghaffar A, Ghanem HM, Ahmed EK, Hassanin OA, Mohamed RG. Ursodeoxycholic acid suppresses the formation of fructose/streptozotocin induced diabetic cataract in rats. Fundam Clin Pharmacol 2018; 32(6): 627–40. doi: 10.1111/fcp.12385 29863796

[27] Wilson RD, Islam S. Fructose-fed streptozotocin-injected rat: an alternative model for type 2 diabetes. Pharmacol Rep 2012; 64: 129–39. doi: 10.1016/s1734-1140(12)70739-9 22580529

[28] Kume WT, Porto EPDJ, Spada ECDL, Lisboa DR, Stachack FFF, Terezo AJ, et al. Acute supplementation of growing rats with Brazil nut flour increases hepatic lipid content but prevents oxidative damage in the liver. J Food Biochem 2021; 45: e13834. doi: 10.1111/jfbc.13834 34180548

[29] Barata PHS, Sarquis IR, Carvalho H, Barros AS, Rodrigues AB, Galue A, et al. Chemoenzymatic synthesis and anti-inflammatory activity of fatty acid amides prepared from *Bertholletia excelsa* (Brazil Nut) triglycerides. J Braz Chem Soc 2020; 31(8): 1–9. doi: 10.21577/0103-5053.20200041

[30] Obafemi TO, Jaiyesimi KF, Olomola AA, Olasehinde OR, Olaoye OA, Adewumi FD, et al. Combined effect of metformin and gallic acid on inflammation, antioxidant status, endoplasmic reticulum (ER) stress and glucose metabolism in fructose-fed streptozotocin-induced diabetic rats. Toxicol Rep 2021; 8: 1419–27. doi: 10.1016/j.toxrep.2021.07.011 34345595 PMC8319514

[31] Nayak Y, Hillemane V, Daroji VK, Jayashree BS, Unnikrishnan MK. Antidiabetic activity of benzopyrone analogues in nicotinamide-streptozotocin induced type 2 diabetes in rats. Sci World J 2014; 2014; 1–12. doi: 10.1155/2014/854267 PMC4274851 25548795

[32] Kotha P, Badri KR, Nagalapuram R, Allagadda R, Chippada AR. Anti-diabetic potential of the leaves of *Anisomeles malabarica* in streptozotocin induced diabetic rats. Cell Physiol Biochem 2017; 43: 1689–702. doi: 10.1159/000484030 29045936

[33] Karl J, Burns G, Engel WD. Development and standardization of a new immunoturbidimetric HbA1c assay. Klinisches Labor 1993; 39: 991–6.

[34] Tietz NW. Clinical guide to laboratory test. 3rd Edition, WB Saunders Company; Philadelphia, 22–23. 1995.

[35] Wei BD, Grossau E, Faderal B. Normal ranges of alpha HBDH, LDH, AP and LAP as measured with substrate-optimated test charges. Med Welt 1975; 26: 387–92. 1121268

[36] Reitman S, Frankel S. A colorimetric method for the determination of serum glutamic oxalacetic and glutamic pyruvic transaminases. Am J Clin Pathol 1957; 28: 56. doi: 10.1093/ajcp/28.1.56 13458125

[37] Friedewald WT, Levy RI, Fredrickson DS. Estimation of the concentration of low-density lipoprotein cholesterol in plasma, without use of the preparative ultracentrifuge. Clin Chem 1972; 18: 499–505. doi: 10.1093/clinchem/18.6.499 4337382

[38] Buege JA, Aust SD. Microsomal lipid peroxidation. Methods Enzymol 1978; 52: 302–10. doi: 10.1016/0003-2697(78)90010-6 672633

[39] Sun M, Zigma S. An improved spectrophotometric assay of superoxide dismutase based on ephinephrine anti-oxidation. Anal Biochem 1978; 90: 81–9. 727489 10.1016/0003-2697(78)90010-6

[40] Rotruckjt RAL, Ganther HF, Swason AB. Selenium: biochemical role as a component of glutathione peroxide. Science 1973; 179: 588–90. doi: 10.1126/science.179.4073.58 4686466

[41] Sinha AK. Colorimetric assay of catalase. Anal Biochem 1972; 47: 389–94. doi: 10.1016/0003-2697(72)90132-7 4556490

[42] Habig WA, Pabst MJ, Jacoby WB. Glutathione transferases. The first step in mercapturic acid formation. J Biol Chem 1974; 249: 7130–9. doi: 10.1016/0003-2697(78)90010-6 4436300

[43] Ganesh M, Mohankumar M. Extraction and identification of bioactive components in *Sida cordata* (Burm.f.) using gas chromatography–mass spectrometry. J Food Sci Technol 2017; 54: 3082–91. doi: 10.1007/s13197-017-2744-z 28974793 PMC5602971

[44] Yu X, Zhao M, Liu F, Zeng S, Hu J. Identification of 2,3-dihydro-3,5-dihydroxy-6-methyl-4H-pyran-4-one as a strong antioxidant in glucose-histidine Maillard reactions. Food Res Int 2013; 51(1): 397–403. doi: 10.1016/j.foodres.2012.12.044

[45] Shareef HK, Muhammed HJ, Hussein HM, Hameed IH. Antibacterial effect of ginger (*Zingiber officinale*) roscoe and bioactive chemical analysis using gas chromatography mass spectrum. Orient J Chem 2016; 32: 817–37. doi: 10.13005/ojc/320207

[46] Igwe OU, Okwu DE. GC-MS evaluation of bioactive compounds and antibacterial activity of the oil fraction from the seeds of *Brachystegia eurycoma* (HARMS). Asian J Plant Sci Res 2013; 3: 47–54.

[47] Junwei L, Juntao C, Changyu N, Peng W. Molecules and functions of rosewood: *Pterocarpus cambodianus*. Arabian J Chem 2018; 11: 763–70. doi: 10.1016/j.arabjc.2017.12.030

[48] Kalaimagal C. Identification of bioactive compounds in flower of *Tabernaemontana divaricata* (L.) using gas chromatography–mass spectrometry analysis. Asian J Pharm Clin Res 2019; 12: 129–32. doi: 10.22159/ajpcr.2019.v12i9.34559

[49] Abdulhafiz F, Mohammed A, Kayat F, Bhaskar M, Hamzah Z, Sanjay PK, et al. Xanthine oxidase inhibitory activity, chemical composition, antioxidant properties and GC-MS analysis of Keladi Candik (*Alocasia longiloba* Miq). Molecules 2020; 25: 2658. doi: 10.3390/molecules25112658 32521624 PMC7321287

[50] Kadhim MJ, Al-Rubaye AF, Hameed IH. Determination of bioactive compounds of methanolic extract of *Vitis vinifera* using GC-MS. Int J Toxicol Pharmacol Res 2017; 9(2): 113–26. doi: 10.25258/ijtpr.v9i02.9047

[51] Shapla UM, Alam SN, Khalil I, Gan SH. 5-Hydroxymethylfurfural (HMF) levels in honey and other food products: effects on bees and human health. Chem Cent J 2018; 12(1): 35. doi: 10.1186/s13065-018-0408-3 29619623 PMC5884753

[52] Vandana CD, Shanti KN, Shantha SL. GC-MS analysis of Callus and leaf extracts of *in vitro* propagated plants of J*usticia wynaadensis* (nees) t. Anderson. Int J Pharm Sci Res 2018; 9(2): 535–43.

[53] Godara P, Dulara BK, Barwer N, Chaudhary NS. Comparative GC–MS analysis of bioactive phytochemicals from different plant parts and callus of Leptadenia reticulata Wight and Arn. Pharmacog J 2019; 11: 129–40. doi: 10.5530/pj.2019.1.22

[54] Selvi ST, Jamuna S, Thekan S, Paulsamy S. Profiling of bioactive chemical entities in Barleria buxifolia L. using GC-MS analysis – a significant ethno medicinal plant. J Ayurvedic Herbal Med 2017; 3: 63–77. doi: 10.31254/jahm.2017.3204

[55] Kakkar S, Narasimhan B. A comprehensive review of oxazole derivatives. BMC Chem 2019; 13(1): 16. doi: 10.1186/s13065-019-0531-9 31384765 PMC6661760

[56] Botzki A, Rigden DJ, Braun S, Nukui M, Salmen S, Hoechstetter J, et al. L-Ascorbic acid 6-hexadecanoate, a potent hyaluronidase inhibitor: X-ray structure and molecular modelling of enzyme inhibitor complex. J Biol Chem 2004; 279: 45990–7. doi: 10.1074/jbc.M406146200 15322107

[57] Begum SMFM, Priya S, Sundararajan R, Hemalatha S. Novel anti-cancerous compounds from *Sargassum wightii*: *In silico* and *in vitro* approaches to test the antiproliferative efficacy. J Adv Pharm Edu Res 2017; 7(3): 272–7.

[58] Keum YS, Chang PPJ, Kwon KH, Yuan X, Li W, Hu L, et al. 3-Morpholinopropyl isothiocyanate is a novel synthetic isothiocyanate that strongly induces the antioxidant response element- dependent Nrf2-mediated detoxifying/antioxidant enzymes *in vitro* and *in vivo*. Carcinogenesis 2008; 29(3): 594–9. doi: 10.1093/carcin/bgm208 17916901

[59] Ghosh G, Panda P, Rath M, Pai A, Sharma T, Das D. GC-MS analysis of bioactive compounds in methanol extract of *Clerodendrum viscosum* leaves. Pharmacog Res 2015; 7(1): 110–13. doi: 10.4103/0974-8490.147223 PMC4285639 25598644

[60] Arora S, Kumar G. Gas chromatography-mass spectrometry (GC-MS) determination of bioactive constituents from the methanolic and ethyl acetate extract of *Cenchrus setigerus* Vahl (Poaceae). Pharma Innov J 2017; 6(11): 635–40.

[61] Sreejith PE, Linu NK, Sasikumar P, Radhakrishnan KV, Sabu M. Phytochemical studies of an endemic and critically endangered hill banana, *Musa acuminata* Colla (AA) ‘Karivazhai’ fruit by GC-MS. J Chem Pharm Res 2016; 8(5): 164–8.

[62] Siswadi S, Saragih GS. Phytochemical analysis of bioactive compounds in ethanolic extract of *Sterculia quadrifida* R.Br. International Conference on Life Sciences and Technology. AIP Conf Proc 2020; 2353: 030098-7. doi: 10.1063/5.0053057

[63] Shoge M, Amusan T. Phytochemical, antidiarrhoeal activity, isolation and characterization of 11-octadecenoic acid, methyl ester isolated from the seeds of *Acacia nilotica* Linn. J Biotechnol and Immunol 2020; 2(1): 1–10. doi: 10.5281/zenodo.3669434.

[64] Vassiliou EK, Gonzalez A, Garcia C, Tadros JH, Chakraborty G, Toney JH. Oleic acid and peanut oil high in oleic acid reverse the inhibitory effect of insulin production of the inflammatory cytokine TNF-α both in in vitro and in vivo systems. Lipids Health Dis 2009; 8: 25. doi: 10.1186/1476-511X-8-25 19558671 PMC2706835

[65] Lopez S, Bermudez B, Pacheco YM, Ortega A, Varela LM, Abia R, et al. Oleic acid: the main component of olive oil on post-prandial metabolic processes. In: Olives and olive oil in health and disease prevention. 2010, Chapter 154, pp. 1385–93. Elsevier Inc. Editors: Victor R. Preedy and Watson Ronald Ross. Spain.

[66] Lattibeaudiere KG, Alexander-Lindo RL. Oleic acid and succinic acid synergistically mitigate symptoms of type 2 diabetes in streptozotocin induced diabetic rats. Int J Endocrinol 2022; 22: 1–10. doi: 10.1155/2022/8744964 PMC8898872 35265127

[67] Velayutham P. Karthi: GC-MS profile of *in vivo*, *in vitro* and fungal elicited *in vitro* leaves of *Hybanthus enneaspermus* (L.) F. Muell. Int J Pharm Pharmaceut Sci 2015; 7(10): 260–7.

[68] Lalitha S, Parthipan B, Mohan VR. Determination of bioactive components of Psychotria nilgiriensis Deb & Gang (Rubiaceae) by GC-MS analysis. Int J Pharm Phytochem Res 2015; 7: 802–9.

[69] Khan IH, Javaid A. Hexane soluble bioactive components of leaf extract of quinoa. J Animal Plant Sci 2022; 32(2): 609–14.

[70] Hussein HJ, Hadi MY, Hameed IH. Study of the chemical composition of Foeniculum vulgare using Fourier transform infrared spectrophotometer and gas chromatography-mass spectrometry. J Pharmacogn Phytother 2016; 8(3): 60–89. doi: 10.5897/JPP2015.0372

[71] Wang T, Liu C, Shu S, Zhang Q, Olatunji OJ. Therapeutic efficacy of polyphenol-rich fraction of *Boesenbergia rotunda* in diabetic rats: a focus on hypoglycemic, antihyperlipidemic, carbohydrate metabolism, antioxidant, anti-inflammatory and pancreato-protective activities. Front Biosci 2022; 27(7): 206. doi: 10.31083/j.fbl2707206 35866393

[72] Consensus Committee, Consensus statement on the worldwide standardization of the hemoglobin A1Cmeasurement: the American Diabetes Association, European Association for the Study of Diabetes, International Federation of Clinical Chemistry and Laboratory Medicine, and the International Diabetes Federation. Diab Care 2007; 30(9): 2399–400. doi: 10.2337/dc07-9925 17726190

[73] Addepalli V, Suryavanshi SV. Catechin attenuates diabetic autonomic neuropathy in streptozotocin induced diabetic rats. Biomed Pharmacother 2018; 108: 1517–23. doi: 10.1016/j.biopha.2018.09.179 30372853

[74] Choudhary M, Aggarwal N, Choudhary N, Gupta P, Budhwar V. Effect of aqueous and alcoholic extract of Sesbania sesban (Linn) Merr root on glycemic control in streptozotocin-induced diabetic mice. Drug Dev Ther 2014; 5: 115–22. doi: 10.4103/2394-2002.139616

[75] Das A, Salloum FN, Filippone SM, Durrant DE, Rokosh G, Bolli R, et al. Inhibition of mammalian target of rapamycin protects against reperfusion injury in diabetic heart through STAT3 signaling. Basic Res Cardiol 2015; 110(3): 31. doi: 10.1007/s00395-015-0486-5 25911189 PMC8554777

[76] Cai L, Wang Y, Zhou G, Chen T, Song Y, Li X, et al. Attenuation by metallothionein of early cardiac cell death via suppression of mitochondrial oxidative stress results in a prevention of diabetic cardiomyopathy. J Am Coll Cardiol 2006; 48: 1688–97. doi: 10.1016/j.jacc.2006.07.022 17045908

[77] Khaki Z, Masoudifard M, Khadivar F, Shirani D, Fathipour V, Taheri M. Serum biochemical and hematological parameters in dogs with benign prostatic hyperplasia (BPH). Iran J Vet Med 2016; 11: 55–62.

[78] Gupta R, Johri S, Saxena AM. Diabetes mellitus; the pandemic of the 21st century. Asian Pacific J Exp Sci 2009; 23(1): 261–8.

[79] Al-Rasheed NM, Al-Rasheed NM, Hasan IM, Al-Amin MA, Al-Ajmi HN, Mohamad RA, et al. Simvastatin ameliorates diabetic cardiomyopathy by attenuating oxidative stress and inflammation in rats. Oxid Med Cell Longev 2017; 2017: 1092015. doi: 10.1155/2017/1092015 29138670 PMC5613468

[80] Wu D, Gao B, Li M, Yao L, Wang S, Chen M, et al. Hydrogen sulfide mitigates kidney injury in high fat diet induced obese mice. Oxid Med Cell Longev 2016; 2016: 2715718. doi: 10.1155/2016/2715718 27413418 PMC4930816

[81] Iyer SS, Cheng G. Role of interleukin 10 transcriptional regulation in inflammation and autoimmune disease. Crit Rev Immunol 2012; 32(1): 23–63. doi: 10.1615/critrevimmunol.v32.i1.30 22428854 PMC3410706

[82] Adoga JO, Channa ML, Nadar A. Kolaviron attenuates cardiovascular injury in fructose-streptozotocin induced type-2 diabetic male rats by reducing oxidative stress, inflammation, and improving cardiovascular risk markers. Biomed Pharmacother 2021; 144: 112323. doi: 10.1016/j.biopha.2021.112323 34656062

[83] Hamdy S, Elshopakey GE, Risha EF, Rezk S, Ateya AI. Abdelhamid FM. Curcumin mitigates gentamicin induced-renal and cardiac toxicity via modulation of Keap1/Nrf2, NF-κB/iNOS and Bcl-2/BAX pathways. Food Chem Toxicol 2024; 183: 114323. doi: 10.1016/j.fct.2023.114323 38056816

[84] Cury MFR, Olivares EO, Garcias RC, Toledo GO, Anselmo NA, Paskakulis LC, et al. Inflammation and kidney injury attenuated by prior intake of Brazil nut in the process of ischemia and reperfusion. Braz J Nephrol 2018; 40(4): 312–18. doi: 10.1590/2175-8239-JBN-2018-0016 PMC6533994 30118536

[85] Wang S, Ding L, Ji H, Xu Z, Liu Q, Zheng Y. The role of p38-MAPK in the development of diabetic cardiomyopathy. Int J Mol Sci 2016; 17(7): 1037. doi: 10.3390/ijms17071037 27376265 PMC4964413

[86] McMullen JR, Sherwood MC, Tarnavski O, Zhang L, Dorfman AL, Shioi T, et al. Inhibition of mTOR signaling with rapamycin regresses established cardiac hypertrophy induced by pressure overload. Circulation 2004; 109: 3050–5. doi: 10.1161/01.CIR.0000130641.08705.45 15184287

[87] He X, Gao F, Hou J, Li T, Tan J, Wang C, et al. Metformin inhibits MAPK signaling and rescues pancreatic aquaporin 7 expression to induce insulin secretion in type 2 diabetes mellitus. J Biol Chem 2021; 297(2): 101002. doi: 10.1016/j.jbc.2021.101002 34303707 PMC8374641

[88] Palmer IS, Herr A, Nelson T. Toxicity of Brazil nuts to rats. J Food Sci 1982; 47: 1595–7. doi: 10.1111/j.1365-2621.1982.tb04990.x

[89] Lincy MLP, Mohan VR, Jeeva S. Preliminary phytochemical screening, gas chromatography mass spectrum and Fourier transform infrared spectroscopy analysis of aerial part of *Maerua apetala* roth (Jacobs). Chem Sci Rev Lett 2015; 4(16): 1275–84.

[90] Tsai MC, Wang CC, Tsai IN, Yu MH, Yang MY, Lee YJ, et al. Improving the effects of mulberry leaves and neochlorogenic acid on glucotoxicity-induced hepatic steatosis in high fat diet treated db/db mice. J Agric Food Chem 2024; 72: 6339–46. doi: 10.1021/acs.jafc.3c09033 38488910 PMC10979445

